# Role of the gut microbiota in anticancer therapy: from molecular mechanisms to clinical applications

**DOI:** 10.1038/s41392-023-01406-7

**Published:** 2023-05-13

**Authors:** Lin-Yong Zhao, Jia-Xin Mei, Gang Yu, Lei Lei, Wei-Han Zhang, Kai Liu, Xiao-Long Chen, Damian Kołat, Kun Yang, Jian-Kun Hu

**Affiliations:** 1 Department of General Surgery & Laboratory of Gastric Cancer, State Key Laboratory of Biotherapy/Collaborative Innovation Center of Biotherapy and Cancer Center, West China Hospital, Sichuan University, Chengdu, China; 2 Gastric Cancer Center, West China Hospital, Sichuan University, Chengdu, China; 3 State Key Laboratory of Oral Diseases, West China Hospital of Stomatology, Sichuan University; Frontier Innovation Center for Dental Medicine Plus, Sichuan University, Chengdu, China; 4 Department of Experimental Surgery, Medical University of Lodz, Lodz, Poland

**Keywords:** Cancer, Cancer therapy

## Abstract

In the past period, due to the rapid development of next-generation sequencing technology, accumulating evidence has clarified the complex role of the human microbiota in the development of cancer and the therapeutic response. More importantly, available evidence seems to indicate that modulating the composition of the gut microbiota to improve the efficacy of anti-cancer drugs may be feasible. However, intricate complexities exist, and a deep and comprehensive understanding of how the human microbiota interacts with cancer is critical to realize its full potential in cancer treatment. The purpose of this review is to summarize the initial clues on molecular mechanisms regarding the mutual effects between the gut microbiota and cancer development, and to highlight the relationship between gut microbes and the efficacy of immunotherapy, chemotherapy, radiation therapy and cancer surgery, which may provide insights into the formulation of individualized therapeutic strategies for cancer management. In addition, the current and emerging microbial interventions for cancer therapy as well as their clinical applications are summarized. Although many challenges remain for now, the great importance and full potential of the gut microbiota cannot be overstated for the development of individualized anti-cancer strategies, and it is necessary to explore a holistic approach that incorporates microbial modulation therapy in cancer.

## Introduction and overview

The human microbiota is dynamically composed of nearly 40 trillion microorganisms with 3000 species, including bacteria, fungi, and viruses, exhibiting variable richness among microbes and diverse constituents among individuals, and is significant for the maintenance of systematic homeostasis and functional stability.^1–5^ The vast majority of members of the human microbiota is located in the gastrointestinal tract (more than 97%), especially in the colon,^1,6^ named the gut microbiota, which has been extensively studied and confirmed to mediate a wide range of physiological functions, such as the development of the immune system and the synthesis of some nutrients.^2,7–9^ Gut dysbiosis occurs when the balance between the microbiota and the human host is disturbed, and manifests as changes in taxonomic composition, metabolic products and secretory vesicles, all of which have been linked to physiological disorders across a broad spectrum of illnesses including cancer.^10–20^

In addition to microorganisms within the digestive tract, the intratumoral microbiota has also drawn increasing attention in the era of precision medicine, since microbes colonizing the tumor microenvironment (TME) may be one of the causes leading to the cancer progression and the discrepancies in the efficacy of cancer therapies among patients.^21–26^ Actually, the local diversity and neoplasm-associated significance of intratumoral microbes were not broadly and deeply investigated until the development of next- and third-generation sequencing in recent years,^27–29^ despite their existence being reported a century ago.^30^ In contrast to its intestinal counterpart, the complex characterization of the intratumoral microbiota is still at an infancy stage, and its roles have not been fully characterized, although some understanding has been gained regarding how it impacts tumorigenesis and therapeutic efficacy. Two main reasons could account for this. The one is, intratumoral microbes are mostly intracellular and are present in cancer cells as well as their surrounding immune cells, which requires more sensitive observation methods to identify the location of intracellular bacteria.^31–33^ The other one is that, the biomass of microorganisms within the TME is very low compared to their gut counterparts, and even just 1 microbial cell may be found in every 10^4^ tumor cells,^34^ which also greatly hinders the investigation of the intratumoral microbiome. Thus, it is necessary to overview some latest mechanistic studies using cutting-edge research methods, which would provide reference for other researchers.

The influences of the gut/tumor microbes on cancer development and treatment, favorable or detrimental, have already been demonstrated in massive mouse experiments. Above all, available evidence from animal experiments has shown that microbes can facilitate the initiation and progression of various types of cancer including gastric cancer,^35,36^ colorectal cancer,^37,38^ hepatocellular carcinoma,^39,40^ breast cancer^20,31^ and pancreatic ductal adenocarcinoma^41,42^. Furthermore, poor response after receiving cancer treatment, including chemotherapy,^43,44^ radiotherapy,^45,46^ surgery and immunotherapy,^47,48^ can be partially ascribed to some microbes, which was also confirmed in mice. The concrete research methods of these preclinical studies are different, but they have a common ground, that is, the pretreatment method for the mice. Specifically, the mice in these studies are inherently germ-free, or they would be pretreated with antibiotic to ensure the consistency of regular microbiota. The research methods are summarized in the Table 1.

**Table 1:** Selective evidence from mouse experiments supporting the effects of human microbes in cancer development

PMID	Article title	Research purposes	Cancer types	Research methods	Findings
**34389621**^35^	Human gastric microbiota transplantation recapitulates premalignant lesions in germ-free mice	To evaluate how gastric microbiota from patients with different gastric disease states affects histopathological features of germ-free mouse stomachs	Gastric Cancer	i. Analyzing gastric microbiota profiles of patients ii. Inoculating biopsies and gastric fluid from patients into mice iii. Analyzing histopathological features of mouse stomachs after 1 month	The gastric microbiota from patients with intestinal metaplasia or gastric cancer selectively colonizes the mouse stomach and induces premalignant lesions, including loss of parietal cells and increases in inflammation foci, in F4/80 and Ki-67 expression, and in CD44v9/GSII lectin expression.
**27876571**^38^	*Fusobacterium nucleatum* Increases Proliferation of Colorectal Cancer Cells and Tumor Development in Mice by Activating Toll-Like Receptor 4 Signaling to Nuclear Factor-κB, and Up-regulating Expression of MicroRNA-21	To identify microRNAs (miRNAs) induced by *F. nucleatum* and evaluate their ability to promote colorectal carcinogenesis in mice	Colorectal cancer (CRC)	i. Incubating CRC cells with *F. nucleatum* and analyzing for miRNA expression patterns, and then incubating cells with miRNAs mimics ii. Injecting *F. nucleatum*-treated cells into nude mice, and then measuring the growth of xenograft tumors iii. C57BL adenomatous polyposis colimin/+, C57BL miR21a-/-, and C57BL mice with full-length miR21a are given *F. nucleatum* by gavage and some mice are given azoxymethane and dextran sodium sulfate, while the controls are given phosphate-buffered saline; then tumors in the intestine are conuted	i. *F. nucleatum* increases proliferation and invasive activities of CRC cells and enhances their ability to form tumors in mice ii. *F. nucleatum* causes these effects by activating Toll-Like receptor 4 signaling to MYD88, leading to activation of the nuclear factor-κB and up-regulation of microRNA-21
**22516259**^39^	Promotion of hepatocellular carcinoma by the intestinal microbiota and TLR4 (Toll-Like Receptor 4)	To explore the role of the gut microbiota in hepatocellular carcinoma development	Hepatocellular carcinoma	i. Inducing hepatocellular carcinoma with diethylnitrosamine and carbon tetrachloride in Tlr4mut and Tlr4WT mice, and then evaluating the contribution of TLR4 in the process of hepatocarcinogenesis ii. Gut-sterilizing mice with antibiotic and reduces systemic lipopolysaccaride levels to examine whether ligands from intestinal bacteria are triggers for the observed TLR4-dependent tumor promotion	TLR4 activation by lipopolysaccharide from the intestinal microbiota contributes to injury- and inflammation-driven tumor promotion but not to tumor initiation
**33408241**^20^	A Procarcinogenic Colon Microbe Promotes Breast Tumorigenesis and Metastatic Progression and Concomitantly Activates Notch and β-Catenin Axes	To explore the effects of enterotoxigenic Bacteroides fragilis (ETBF) in the initiation and development of breast cancer	Breast cancer	i. Treating normal breast epithelial cells and breast cancer cells with Bacteroides fragili toxin (BFT) and conducting functional experiments ii. Pretreating breast cancer cells with BFT followed by mammary gland implantation in mice, and histological characteristics of tumor sections are investigated iii. Tumors formed in the mice are subjected to RNA-seq analysis to uncover the underlying molecular changes iv. Colonization of ETBF in the mouse gut or mammary duct to determine whether it affects mammary tumorigenesis and metastatic progression.	i. Multiple human breast cancer cell lines, upon exposure to BFT, showed increased invasion and migration potential ii. BFT exposure enables the formation of multifocal breast tumors with elevated stemness character in mouse iii. β-Catenin and Notch1 pathways are involved in mediating the impact of BFT in the breast
**29567829**^52^	The Pancreatic Cancer Microbiome Promotes Oncogenesis by Induction of Innate and Adaptive Immune Suppression	To explore the mechanisms of the pancreatic cancer microbiome in the oncogenesis	pancreatic cancer	i.Performing 16 S rRNA gene sequencing on tumor tissues from patients ii. Ablating gut bacteria in KC mice with antibiotic, then repopulating them with feces from either WT mice or KPC mice	i. A markedly greater presence of bacteria in both mouse and human pancreatic ductal adenocarcinoma compared with normal pancreas is found ii. Pathogenic bacteria in the pancreatic cancer promote the oncogenesis

The microbiota in the alimentary tract and TME can be considered, to some extent, as a dynamic system whose internal compositions are both interconnected and relatively independent. Specifically, tumors and other causes of the disruption of the intestinal mucosal barrier may provide access for the gut microbes, resulting in their switching to intratumoral microbes directly involved in the development of cancer.^49^ Thus, it is not surprising that both gut and intratumoral microbes can exert cancer-promoting effects.^41,50–55^ In the early stages of many digestive cancers, alterations at the cellular level in the alimentary tract often shows up as gut dysbiosis, followed by the pro-carcinogenic effects of various bacterially secreted oncogenic molecules. More importantly, diverse molecular mechanisms by which pathogenic microbes contribute to tumorigenesis^56,57^ have been found over the past few years. For example, a genotoxin called colibactin generated by pathogenic *pks*^*+*^ *Escherichia coli* can alkylate DNA, which may be involved in the development and progression of colon cancer.^58,59^ In addition, other typical tumor-related bacteria, such as *Fusobacterium nucleatum* and *Helicobacter pylori* (*Hp*), can promote cancer through a complex set of mechanisms, including chronic inflammation, DNA damage, and the activation of oncogenic pathways.^60–65^

The carcinogenic and anti-cancer mechanisms of microbes are extremely intricate, and only a tip of the iceberg has been thoroughly probed. Nonetheless, the potential of microbial strategies for cancer therapy have been demonstrated in many clinical trials. Specifically, human microbiota can be modified to boost the host response to the existing anti-cancer therapies and minimize the corresponding adverse toxicities and reduce drug resistance in immunotherapy, chemotherapy, cancer surgery, and radiation therapy, and specific interventions targeting the microbiota include (but not limited to) diet-based interventions, prebiotics, probiotics, postbiotics, targeted antibiotic approaches, and fecal microbiota transplantation (FMT).^66,67^ For example, clinical research results generated by Zheng et al. indicated that probiotic compound can significantly relieve inflammation, enhance immunity, and promote recovery in patients with gastric cancer after gastrectomy, and thus it may serve as an adjuvant treatment for gastric cancer in the future.^68^

One of the ultimate purposes of basic research is for clinical practice, and thus follow-up clinical trials based on the preclinical findings need to be designed and conducted as much as possible. Pan and colleagues have found that *Clostridium butyricum* strains MIYAIRI 588 (CBM588) can ameliorate acute pancreatitis by maintaining intestinal homeostasis in mice,^69^ and a recently published clinical trial showed that CBM588 can obviously prolong progression-free survival (PFS) in patients with metastatic renal cell carcinoma treated with nivolumab-ipilimumab.^70^ Against this background, animal experiments were conducted to preliminarily explore the properties of CBM588 in a general direction, and another team subsequently confirmed the anti-cancer effect of CBM588 through clinical trial based on the previous findings. Thus, combining basic mechanistic studies with corresponding clinical trials is essential, which will be conducive to moving the field of microbiology-oncology gradually forward from bench to bedside. However, the road that combines clinical trials with the basic studies is full of challenges, which presents a great obstacle to clinical translation of microbial strategies for cancer therapy. For example, unlike animal models, the baseline characteristics of the gut microbiota among human subjects are hard to keep consistent artificially, which dramatically impedes the design and implementation of corresponding clinical trials.

In this review, the initial clues of molecular mechanisms regarding the carcinogenic effects of gut and tumor microbes are first summarized, based on which the significance of microbes for conventional cancer treatment is also addressed. In addition, current and emerging microbial interventions for cancer therapy as well as their clinical applications are also highlighted, with emphasis on the latest major studies on boosting the efficacy of traditional cancer treatment and reducing its side effects via microbial strategies, which may provide insights into the formulation of individualized therapeutic strategies for cancer therapy. Finally, the authors’ perspectives regarding the outlook and challenges of microbial strategies in basic studies and clinical translation are summarized.

## Contributions of microbes from different dimensions in cancer development

Gut dysbiosis refers to a less stable and diverse and more pathogenic microbiota that is reshaped when the sophisticated balance of the microecosystem in the gastrointestinal tract is disturbed, which contributes to a variety of pathological conditions by adversely affecting the physiological processes of the host.^71,72^ More importantly, pathogenic microbes may have a harmful impact in the development and treatment of cancer.^73,74^ To gain a better understanding of the molecular mechanisms, the influence of microbes on normal epithelial tissue and tumor microenvironment should first be explained before further discussion.

**Fig. 1:**
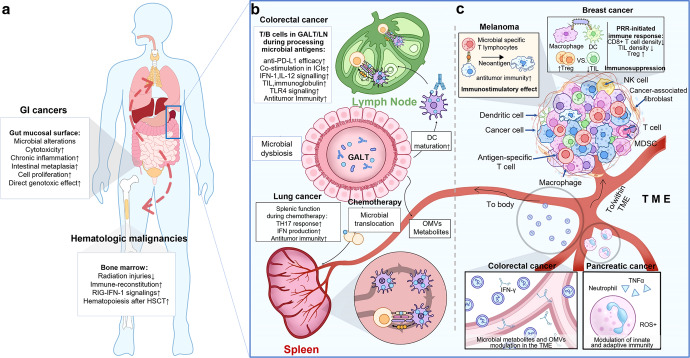
Interactions between the gut microbiota and cancer development. The gut microbiota can interact with cancer through various patterns, one of which is contact-dependent interactions that occur locally at mucosal surface or within primary lymphoid organs including the bone marrow and the thymus (**a**), and secondary lymphoid organs including the GALT, lymph nodes and the spleen (**b**) or the TME (**c**). Another one is contact-independent interactions which present systematically via microbial metabolites and OMVs in circulation (**c**). Specifically, **a** Gut microbes can interact directly with the gastrointesinal tract mucosal surface, resulting in genotoxic effect, epithelial cell proliferation, loss of cellular polarity, intestinal metaplasia; the hematopoiesis of the thymic and bone marrow could be stimulated by microbiota via RIG-IFN-1 signaling especially after HSCT, thus making radio-protective effect in the radiotherapy. **b** Gut microbes and their metabolites or OMVs interact with the GALT, LN and spleen, through the T cells and dendritic cells regulations via various patterns, such as enhancement of the TH17 response, IFN production, antigen presentations and signaling of IFN-1, IL-12, TLR4. **c** Microbes both in the gut and tumor could exert influence on the TME, either with immunostimulatory effect via presenting microbial specific antigen to the T cells, or with immunosuppressive effect via regulating the balance of the Treg and TILs. Besides, microbial modulation of the TME exemplified are means by which microbiome-secreted metabolites, cargo-carrying OMVs, or may induce a complex array of immunomodulatory actions via circulation. Microbial secreted moieties can impact the TME innate immune response, by modulating attraction and activation of innate immune cells such as neutrophils, producing TNFα and ROS to combat tumorigenesis, and influence the adaptive immune response by co-stimulating T cells mentioned above. (HSCT hematopoietic stem cell transplant, DC dendritic cell, GALT gut-associated lymphoid tissues, LN lymph node, TLR4 Toll-like receptor 4, TME tumor microenvironment, CTL cytotoxic T lymphocyte, NK cell natural killer cell, OMVs outer membrane vesicles, SCFAs short-chain fatty acids, TIL tumor-infilrating lymphocyte, PRR pattern recognition receptor, MDSC myeloid-derived suppressor cells, ROS reactive oxygen species, TNF α tumor necrosis factor α)

The microbes can impact cancer in various manners,^75,76^ one of which is contact-dependent effects that occur locally at the mucosal surface or in the TME. Another is contact-independent effects, which are systematically present via microbial metabolites and outer membrane vesicles (OMVs) in circulation. (Fig. 1) The concept of contact-dependent effects is well understood, but the mechanisms involved in contact-independent effects may be slightly more complex. Herein, contact-independent effects are defined as a biological phenomenon in which gut microbiota-derived detrimental molecules enter the bloodstream through capillaries, directly facilitating the development of distant cancer, or indirectly promoting its progerssion by weakening the antitumor immunity of the host. For example, lipoteichoic acid (LTA) and deoxycholic acid (DCA), a cell wall component and a metabolite of gram-positive gut bacteria, respectively, have been corroborated to promote the development of hepatocellular carcinomas after translocation into the liver through the enterohepatic circulation,^77,78^ which is typical contact-independent effect of gut microbes on cancer. In this chapter, we will depict the effects of microbes in cancer development from two different dimensions.

### The effects of microbes on intestinal mucosal surface

Normally, the gut microbiota in healthy human body is generally considered as beneficial, but some luminal microbes may pose a potential threat to the host. Compared with healthy individuals, a variety of microbes are more frequently observed in the stool and on the gut mucosa of patients with gastrointestinal tumors,^79–81^ and in vivo experiments have shown that microorganisms play a paramount role in carcinogenesis.^82,83^ However, we still know little about the direct impacts of microbes on normal gut epithelial cells (ECs). In this section, we will emphasize how certain bacteria within the alimentary tract directly affect ECs and trigger malignant transformation.

When investigating the effects of microorganisms on cancer initiation, the first issue we should determine is whether they cause DNA damage and abnormal gene mutations in ECs. *H. pylori* plays a nonnegligible role in the process of gastric cancer initiation, and one of its main mechanisms inducing gastric carcinogenesis is causing DNA damage via oxidative stress in the gastric mucosa.^84^ Prior to direct contact with ECs, *H. pylori* secretes proteases and phospholipases to degrade the mucus layer on the mucosal surface in the stomach, which enhances *H. pylori* adherence.^85^ Subsequently, cytotoxin-associated gene A (CagA), one of the main virulence factors generated by *H. pylori*, upregulates the levels of spermine oxidase (SMO) that metabolizes the polyamine spermine into spermidine and generate H2O2, which would cause apoptosis and DNA damage of ECs; thus, a subpopulation of epithelial cells gradually becomes resistant to apoptosis and is at high risk for malignant transformation.^84,86^ To sum up, some pathogenic microorganisms have the ability to colonize the mucosal surface of digestive tract and can increase the risk of malignant transformation in ECs through DNA damage mechanism.

Bacteria may also induce epithelial inflammation and the disruption of the mucosal barrier, both of which are linked to the carcinogenesis. *F. nucleatum*, one of the resident bacteria constituting the oral microbiota, has been confirmed to accelerate the initiation, progression and metastasis of colorectal cancer (CRC) in recent studies,^87,88^ and its impact on intestinal epithelial cells has been increasingly identified. Engevik et al. found that *F. nucleatum subsp. polymorphum* can release OMVs to activate TLR4 and NF-κB on colonic epithelial cells, which ultimately stimulates the production of downstream proinflammatory factors associated with intestinal inflammation.^89^ Remarkably, the proinflammatory effects were absent in the context of an intact gut microbiota, which implicitly indicated the significance of a normal gut microbiota. Additionally, OMVs secreted from *F. nucleatum* can also adversely alter the epithelial homeostasis by impairing the intestinal mucosal barrier in ulcerative colitis.^90^ Because chronic inflammation and disruption of the intestinal mucosal barrier can increase the risk of tumorigenesis,^91,92^ we assume that the effects of *F. nucleatum* on ECs and mucosal barrier are the significant causes that induce the transformation of precancerous conditions to cancer.

### The interactions between microbes and the tumor microenvironment (TME)

The TME is the internal environment upon which the existence and proliferation of tumor cells depend, and it contains a variety of cells, including tumor cells, stromal cells, and immune cells (such as T lymphocytes, B lymphocytes, natural killer cells, and tumor-associated macrophages), as well as a dense network of microvessels.^93^ Apart from the regular components, growing evidence has shown that bacteria reside in the cancer cells and immune cells within the TME,^31,32^ which has an underlying impact on the biological phenotype of cancer cells and local immune microenvironment within the TME.

On account of some inherent characteristics in tumors, the TME is well-suited for the invasion, colonization and growth of microbes. First, during the process of carcinogenesis, many angiogenic factors released by tumor cells induce vascularization,^94^ which is conducive to the invasion of distant microbes into TME. Additionally, tumor is generally characterized by inherent immune privilege,^95^ and microbes within the TME can also serve as immune inhibitors.^96^ This combined immunosuppressive phenomenon is favorable for the colonization and growth of intratumoral microbes.

Moreover, the conditions within the TME, such as local oxygen concentration, can influence the composition of tumor microbiota. For example, hypoxic and even anoxic inner regions is a characteristic feature of many solid tumors arising from an imbalance between oxygen supply and consumption,^97,98^ which is accompanied by the resultant accumulation of microaerophilic and anaerobic bacteria in the TME, such as *Bacteroides fragilis* and *Enterococcus faecalis* in CRC,^99^ and the relative abundance of aerobic bacteria in the tumor may be lower. Notably, there is spatial heterogeneity of oxygen concentration within tumor;^100^ however, it is unclear whether this uneven oxygen distribution would lead to diverse microbial members across different regions within the TME, which needs further study. Additionally, distinct microbiome compositions have been discovered across different tumor types,^32^ which may be a result from multifaceted effects, and more and further investigation is needed.

Intratumoral bacteria may affect the phenotype of cancer, such as enhancing the metastatic ability of malignant cells. Using the murine spontaneous breast-tumor model, Fu and colleagues found that significant amounts of tumor-resident bacteria reside in the cytoplasm of cancer cells and that these bacteria can facilitate the metastasis in breast cancer by reorganizing the cellular cytoskeleton and enhancing resistance to mechanical stress.^31^ Additionally, a conserved intracellular bacterial profile represented by *Enterococcus* and *Streptococcus* was also detected in human breast cancer, which could metastasize to distant sites with cancer cells.^31^ Thus, the two findings collectively suggest that microbes inhabit in human breast cancer, and the bug may promotes cancer progression. Similarly, *F. nucleatum* can reinforce the metastatic potential of CRC through various complex mechanisms.^101,102^ Additionally, other biological behaviors of CRC, including proliferative and invasive abilities, can also be enhanced by *F. nucleatum*.^38,103^ In the future, further clinical trials targeting the microbes within breast cancer should be designed and conducted, which may reduce breast cancer metastasis.

Additionally, the bacterial signals may promote cancer development by inhibiting local antitumor immunity.^96^ For example, *F. nucleatum* within the CRC is negatively associated with the density of CD3^+^ T-cell infiltrated in the TME, which relates to the downregulation of antitumor adaptive immunity.^104^ One thing to note, the bacteria that induce immunosuppression in the TME may actually be derived from the intestinal tract and the oral cavcity.^52,105^ The effects of intratumoral microorganisms on tumors are extremely complicated, and sometimes the same bacteria may not have identical impact on the same tumor. In colorectal cancer with low levels of microsatellite instability (MSI), *F. nucleatum* is positively correlated with tumor-infiltrating lymphocytes.^106^ Therefore, a bold conclusion can be drawn that other host factors besides the level of MSI may also influence the role of microorganisms within the TME, but more research is needed.

The effects of bacteria on the TME can be realized specifically through their OMVs or metabolites. OMVs constitute a crucial microbial delivery system that allows microbes to transfer their virulence factors, proteins and genetic materials in the systemic circulation. More importantly, microbe-derived cargos within OMVs can adversely reshape the TME. For example, OMVs released by *H. pylori* harbor active CagA that activates TLR and NF-κB pathways in gastric cells, which reinforces the inflammation and cell proliferation associated with carcinogenesis.^107,108^ In addition, certain microbial metabolites may be involved in the formation of the TME. DCA is a secondary bile acid produced by gut microorganisms after metabolizing primary bile acids. Song et al. suggested that DCA could facilitate vasculogenic mimicry and epithelial-mesenchymal transition (EMT) through activating vascular endothelial growth factor receptor 2, which is critical for the malignant transformation of intestinal epithelium.^109^

Other types of microbes such as fungal have also been found in the TME. For example, *Malassezia* species has been discovered in pancreatic ductal adenocarcinoma, and the glycans on its cell wall can bind to mannose-binding lectins to activate the complement cascade, which promotes tumor progression.^41^ Additionally, Dohlman and colleagues have confirmed the presence of *Candida* species in gastrointestinal tumors, which may correlate with worse survival outcomes, pro-inflammatory gene expression, and metastasis in cancer patients.^34^ Notably, relationship between fungal and bacterial communities within the TME is generally peace rather than competition;^33^ however, whether this harmonious relationship means a synergistic cancer-promoting effect remains unclear.

## Mechanisms of microbes in tumorigenesis

**Fig. 2:**
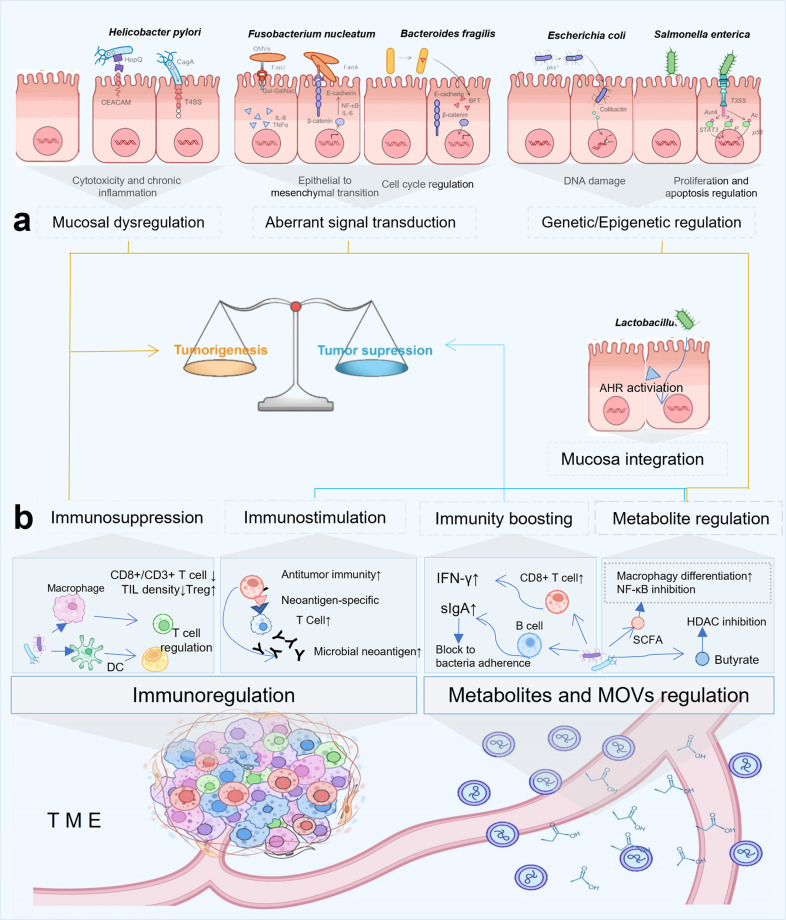
Mechanisms of microbial tumorigenesis and tumor suppression. **a** Mechanisms of microbes instigating tumorigenesis and tumor suppression in the gut: (1) mucosal dysregulations: For example, the virulence factor CagA secreted by *H. pylori* can inject into the mucosal cells via T4SS with the combination of CEACAM and HopQ, thereby promoting cell proliferation and improve the transformation rate of tumor cells. (2) aberrant signals transduction: For example, Fap2 extracted from *F. nucleatum* can mediate tumor progression via binding to the Gal-GalNAc, and OMVs from *F. nucleatum* can also stimulate colonic epithelial cells producing TNF and trigger IL-8 signaling; FadA, another pathogenic factor from *F. nucleatum*, can interact with E-cadherin on the epithelial cells and activate NF-κB pathway via Wnt/β-Catenin signaling, resulting in tumorigenesis. (3) DNA damage and induced genetics/epigenetics alteration: e.g. T3SS of *Salmonella enterica* can bind the effector protein AvrA and cyclomodulin-like protein typhoid toxin, promoting tumorigenesis genetically and epigenetically, through genotoxin-mediated mutagenesis. Specifically, AvrA promotes cell proliferation, differentiation and inhibits cell-cycle arrest via JAK/STAT, Wnt/β-catenin or acetyltransferase-targeted p53 pathway, collectively resulting in tumorigenesis. *Escherichia coli* can induce DNA damages via a secreted genotoxin, colibactin, which can break the DNA doublestrand and crosslinks. (4) immune suppression: For example, intratumoral microbes can reduce immunosurveillance effect via PRR ligation with larger proportions of Tregs and lower ratio of TILs, e.g. CD8 + T lymph cells, thus inducing tumor proliferation and metastasis. **b** Mechanisms of microbes instigating tumorigenesis and tumor suppression in the TME: (1) Immunity boosting: For example, bacterial metabolites can elevate IFN-γ-producing CD8 + T cells, enhance the therapeutic effect of ICIs in mouse models, and SCFAs from gut bacteria can stimulate the formation of mucus from goblet cells, inhibit NF-κB activation, elicit inflammation signal and produce IL-18, promote the secretion of sIgA from B cells, thus boosting the immunity. (2) Metabolite regulation in anti-cancer activity: For example, SCFAs, such as butyrate, from commensal bacteria can induce the differentiation of macrophage and increase the antibacterial activity of macrophage, partly through inhibition of HDAC3 activity, which plays a role in glycolysis and autophagy, thus regulating the tumorigenesis and tumor suppression. (CagA the cytotoxin-associated gene A, T4SS the type 4 secretion system, CEACAM carcinoembryonic antigen-related cell adhesion molecules, Hop Q outer membrane adhesion, OMVs outer membrane vesicles, TNF tumor necrosis factor, IL-8 interleukin-8, Treg regulatory T cell, TILs tumor infiltrating lymphocytes, TME tumor microenvironment, IFN- γ interferon γ, T3SS the type 3 secretion system, PRR pattern recognition receptor, ICIs immune checkpoint inhibitors, sIgA secretory IgA, SCFAs short-chain fatty acids, HDAC histone deacetylase)

Cancer-promoting bacteria may participate in the process of oncogenesis through a variety of different molecular pathways, and four main mechanisms are summarized here (Fig. 2): (1) DNA damage and epigenetics alterations; (2) interference with the DNA damage response (DDR) (3) abnormal signaling pathways; and (4) immune suppression.

### Inducing DNA damage and epigenetics alterations

In essence, cancer is nothing other than a disease of genes. Thus, if a microbe is involved in cancer initiation, it would probably give rise to genetic mutations represented by DNA damage in normal cells.^110^ A range of bacteria have the ability to induce DNA damage in host cells either directly through the effects of genotoxins or indirectly by activating cell-autonomous responses, which may be involved in the cancer initiation.

In a recent study, scholars from Yale University have found that bacterial strains isolated from patients with inflammatory bowel disease (IBD) exhibited DNA-damaging activities associated with malignant transformation from IBD to colon cancer.^111^ More importantly, a previously unexplored family of genotoxic small molecules termed the indolimines generated by Morganella morganii was discovered, which could increase the intestinal permeability and exacerbate colon tumorigenesis in gnotobiotic mice.^111^ Similarly, colibactin, a genotoxin expressed by *pks+* *E. coli*, was also shown to induce DNA damage in colonic epithelial cells and correlate with faster cancer onset in patients with familial adenomatous polyposis, a precancerous stage for colon cancer.^12^ Mechanistically, colibactin can lead to alkylation and interstrand crosslinks after binding to DNA, which interfere with DNA replication and transcription, resulting in DNA double-strand breaks.^112^ Intriguingly, colibactin has also been detected in *Klebsiella pneumoniae*, *Enterobacter aerogenes* and *Citrobacter koseri* isolates,^113^ which implies possible carcinogenic effects of these bacteria. Other major genotoxin-producing bacteria include *H. pylori*,^114^ *Bacteroides fragilis*,^115^ *Salmonella enterica*,^116^ etc.

In addition to the direct effects induced by genotoxins, DNA damage can also be indirectly induced by infected cell-autonomous mechanisms in response to the presence of bacterial pathogens or their byproducts. Free radicals, such as reactive oxygen species (ROS), can be produced by infected host cells, and are also important DNA damaging agents because they can result in the base oxidation as well as the production of abasic sites (known as so-called AP sites) and DNA strand breaks. Pathogens including *Chlamydia trachomatis*,^117,118^ *B. fragilis*^119^ and *H. pylori*^120^ can trigger the production of ROS in infected cells, and the corresponding mechanisms have been thoroughly investigated. For example, similar to the *H. pylori*-secreted CagA described above, *B. fragilis* toxin can also upregulate SMO and result in SMO-dependent production of ROS, which induces DNA damage.^119^ More importantly, animal experiments corroborated that inhibiting SMO could significantly reduce ETBF-induced colon tumorigenesis, suggesting the vital role of this pathway in tumorigenesis.^119^

Apart from the production of ROS, other cell-autonomous responses inducing DNA damage can also be elicited by the bacteria. For example, *H. pylori* can induce DNA double-strand breaks (DSBs) after adhering to host cells, through binding the bacterial type IV secretion system to host cell integrin β1 and subsequent activation of NF-κB (nuclear factor-κB) signaling.^121^ Subsequently, DSBs are induced by the nucleotide excision repair endonucleases XPF and XPG, which are recruited to chromatin together with the NF-κB p65 subunit.^121^

Additionally, microbes may be involved in cancer development via epigenetic mechanisms. Epigenetic alterations mainly encompass the methylation of DNA, the posttranslational modification of histones, chromatin remodeling and regulation by noncoding RNAs, of which the methylation of DNA is the most well-explored. CRC development is closely linked with hypermethylation, which can slience tumor suppressor gene.^122^ Recently, Xia and colleagues found that the *Hungatella hathewayi* and *F. nucleatum* enriched in CRC were significantly associated with *CDX2* and *MLH1* (both are antioncogenes) promoter hypermethylation, respectively, through which the bacteria may drive intestinal tumorigenesis.^123^ Mechanistically, in vitro and in vivo experiments have demonstrated that both bacteria could upregulate DNA methyltransferase, which is required for hypermethylation.^123^

### Interference with the DNA damage response (DDR)

The human genome constantly suffers from damage caused by exogenous factors including pathogenic microbes and endogenous genotoxic stress from cellular physiological processes such as DNA replication stress.^124,125^ Thus, human cells have evolved elaborate mechanisms, collectively termed the DNA damage response (DDR),^126^ to identify detrimental DNA mutations and repair impaired DNA sites, the normal outcomes of which include apoptosis as well as transient cell cycle arrest promoting DNA repair or cellular senescence.^124^ However, microbes not only induce DNA damage but also interfere with the DDR to hinder the repair of damaged DNA, both of which promote the passage of detrimental mutations to their progeny cells and may be associated with oncogenesis. When DSBs occur, DDR is initiated by the MRN complex, which is composed of MRE11, RAD50 and NBS1.^127^ Subsequently, ataxia-telangiectasia mutated kinase (ATM) is recruited and activated by the MRN complex, phosphorylating downstream proteins and subunits of the protein complex, which plays a pivotal role in the repair of damaged DNA.^127–129^

*C. trachomatis*, a pathogen associated with cervical and ovarian cancer, contributes to DNA damage by inducing the production of ROS as discussed above.^117,118^ More importantly, it can interfere with the DDR in infected host cells. Specifically, *C. trachomatis* inhibits the activation and recruitment of MRE11, ATM and 53BP1(p53-binding protein 1, a key player in orchestrating the choice of DNA repair pathway) at impaired DNA sites, as well as the activation of CHK1- and CHK2-mediated cell-cycle checkpoints, both of which may predispose host cells to malignant transformation.^118^ Likewise, *H. pylori* is a representative microbe that not only induces DSBs but also interferes with various DDR pathways. For example, *H. pylori* can elicit decreased expression of MutS and MutL at the protein level,^130^ both of which are components of the DNA mismatch repair system, and the aberrant upregulated expression of AID (activation-induced cytidine deaminase)^131^ associated with a high frequency of TP53 mutation. Consequently, the combination of these two effects of *H. pylori* may lead to higher point mutation rates and increased risk of carcinogenesis.

Additionally, *H. pylori* suppresses homologous recombination (HR), an error-free DNA damage repair pathway, while promoting non‑homologous end‑joining (NHEJ), an error-prone pathway, both of which are for DSBs.^132,133^ Specifically, *Hp* infection can inhibit the expression of MRE11 and downstream proteins RAD54 and RAD51, all of which are responsible for the regulation of HR, and it can facilitate the recruitment of 53BP1, which drives NHEJ.^133–136^ NHEJ is one of the major pathways for DSB repair, and plays a significant role in the maintenance of genome integrity via template-independent repair throughout the entire cell cycle. However, compared with HR, NHEJ induces chromosomal and genomic instability, especially in the context of defects in other DSB repair pathways, and an overactive NHEJ pathway may be associated with the development of malignancies.^137,138^ Thus, one of the *Hp-c*arcinogenic mechanisms may be the inhibition of HR and the promotion of NHEJ.

The p53 protein is an important regulator of the DDR, promoting either the apoptosis or repair of damaged cells and is kept at a low level under unstressed states via the proteasome instructed by the E3 ubiquitin ligase MDM2.^139^ Normally, if DNA damage occurs, p53 will be phosphorylated to drive follow-up responses, such as cell cycle arrest.^140,141^ However, Hassin et al. found that *H. pylori* can induce the degradation of p53 to interfere with the DDR process.^142^ Specifically, *Hp*-secreted CagA interacts with apoptosis-stimulating protein of p53 (ASPP2), a protein activating p53 following DNA damage and consequently triggering apoptosis, and relocates it to an area near the plasma membrane, which confines p53 to the cytoplasm and consequently results in the MDM2-mediated proteasome-involved degradation of p53.^143^ More importantly, the degradation of p53 would increase the resistance of infected cells to apoptosis, thereby enhancing the colonization of *Hp* and predisposing these epithelial cells to cancerous transformation.^143^

### Triggering aberrant signaling pathways

In addition to accelerating carcinogenesis by interfering with DDR pathways, microbes can also adversely impact other signaling pathways to promote cancer. Wnt/β-catenin signaling is a vital and highly conserved pathway controlling numerous biological processes, such as cell fate determination during embryonic development.^144^ However, aberrant activation of Wnt signaling has been demonstrated to be closely linked to many biological processes of cancers, including initiation and progression.^145,146^ Sufficient evidence has shown that bacteria can modulate the Wnt pathway, thereby triggering malignant transformation. Fusobacterium adhesin A (FadA) is a virulence factor generated by generated by *F. nucleatum*, and it can modulate E-cadherin/β-catenin signaling to promote colorectal carcinogenesis.^88^ Specifically, FadA binds to E-cadherin on the membrane, leading to the phosphorylation and internalization of E-cadherin, which is accompanied by increased β-catenin release and translocation into the nucleus due to the degradation of the E-cadherin/β-catenin complex, resulting in the aberrant activation of Wnt signaling associated with various cancers.^88,147^ Similarly, a virulence factor termed BFT secreted by ETBF can also activate Wnt signaling by cleaving E-cadherin.^148,149^

MAPK (mitogen-activated protein kinases) belongs to the family of serine-threonine kinases, which may be activated to promote carcinogenesis by certain bacteria. There are three kinds of crucial kinases in the MAPK family: extracellular signal-regulated kinase (ERK), JUN N-terminal kinase (JNK) and the stress-activated protein kinase p38 MAPK.^150^ *Hp*-derived CagA can trigger the ERK signaling cascade through interaction with growth factor receptor-bound protein 2 (GRB2), thereby activating T cell factor (TCF).^151^ Subsequently, TCF promotes the expression of induced myeloid leukemia cell differentiation protein 1 (MCL1), which may prevent the apoptosis of gastric epithelial cells.^151^ In addition, *Salmonella Typhi*, one of the risk factors for gallbladder carcinoma,^152^ can also activate the MAPK pathway and AKT pathway, which may accelerate the transformation of cells with silent p53 and overactive MYC.^153,154^

### Eliciting immunosuppressive effects

The human immune system has a function termed immunosurveillance, whereby aberrant cells can be recognized and eliminated. Therefore, cancer cells must escape from detection and killing by the immune system for the tumorigenesis.

Recent studies have corroborated that bacteria can protect cancer cells from immunosurveillance, which may be linked to the development of cancer. For example, *F. nucleatum* can inhibit the attack of natural killer (NK) cells on tumor cells by binding TIGIT, an inhibitory receptor on human NK cells and various T cells, via the fusobacterial Fap2 protein.^155^ Additionally, gut microbes promotes pancreatic ductal adenocarcinoma by decreasing the intratumoral infiltration and activity of NK cells.^42^ Furthermore, *F. nucleatum* can selectively recruit tumor-infiltrating myeloid-derived suppressor cells (MDSCs), which may promote intestinal tumorigenesis by suppressing the immune response.^156,157^ More importantly, MDSCs may contribute to the formation of premetastatic niches^158,159^ and metastases by infiltrating primary tumors.^160,161^ Therefore, based on the above findings, we can conclude that *F. nucleatum* may indirectly facilitate metastasis by promoting the accumulation of MDSCs.^162^

Likewise, gut gram-negative bacteria/lipopolysaccharide direct hepatocytes to recruit MDSCs in liver in the context of benign liver disease or colitis that disrupts intestinal barrier, and thus promoting liver cancer by forming an immunosuppressive microenvironment.^163^ *H. pylori* also helps precancerous cells escape from immunosurveillance in the process of malignant transformation. For example, *H. pylori* can induce the expression of programmed death ligand 1 on gastric epithelial cells via the Sonic Hedgehog signaling pathway, whereby *Hp*-infected cells may escape immunosurveillance and progress to gastric cancer cells.^164^

Gut microbiota-derived metabolites also suppress anticancer immunity. Hezaveh et al. found that indole compounds, tryptophan metabolites produced by *Lactobacillus*, can activate the aryl hydrocarbon receptor in tumor-associated macrophages, which inhibits the intratumoral infiltration of TNFα + IFNγ + and CD8 + T cells in the pancreatic ductal adenocarcinoma and correlates with rapid disease progression and mortality.^165^

In addition to bacteria, pathogenic fungi also adversely regulate immunosurveillance. Rieber and colleagues have found that *Aspergillus fumigatus* and *Candida albicans* can induce MDSCs through the PRR Dectin-1 and its downstream adaptor protein CARD9, which functionally suppress T and NK cell responses.^166^

In the process of carcinogenesis, escape from immunosurveillance is an essential link. Ample evidence has substantiated that factors besides mutated cells themselves, such as the microbes discussed in this article, also suppress immunosurveillance against abnormal cells and contribute to malignant transformation.

## Mechanisms of microbes in tumor suppression

Microorganisms not only promote cancer, but also inhibit its occurrence and progression through the following two mechanisms: direct killing effects on tumor cells and positive immunoregulatory effects.

### Direct tumor-suppressive effects

As discussed above, bacterial genotoxins can initiate and promote cancer. However, some bacterial toxins also exhibit targeting property against cancer cells and thus may serve as underlying anticancer agents.^167^ *Clostridium perfringens* enterotoxin (CPE) is the virulence factor that causes the symptoms of *C. perfringens* type A food poisoning,^168^ while it also fights cancer cells by binding to transmembrane tight junction proteins claudin-3 and −4 that are highly expressed in human cancers, including breast,^169^ prostate^170^ and colon cancer.^171^ Mechanistically, the interaction between CPE and claudins triggers the formation of pore complex in the plasma membrane, resulting in the loss of osmotic equilibrium between intracellular and extracellular fluids and cell death.^172^ Other bacteria that have been identified as direct antitumor microbe include *Pseudomonas aeruginosa*, *Salmonella typhimurium* and *Clostridium difficile*, all of which generate toxins that display anticancer activity.^173–176^ Therefore, future chemotherapy agents may be developed from the toxins extracted from these microorganisms or their attenuated derivatives. Because bacterial toxins are generally toxic to normal cells, modification of the virulence factors with genetic engineering techniques is needed to overcome systematic toxicity in most cases.

### Positive immunoregulatory effects

Some microbes can prevent and suppress cancer via immune mechanisms. On the one hand, normal gut microbiota is critical for the development of host immune system, and its absence would result in the structural and functional disability of the immune system,^7^ which may be associated with cancer initiation. For example, gut microbiota can promote the maturation of lymphoid organs and the differentiation of immune cells, which reflect the effects of microorganisms on the structure and function of immune system, respectively.^177^ Lymphoid tissue is divided into the central lymphoid organs and the peripheral organs. The central lymphoid organs are the sites in which B- and T-lymphocytes are generated, including bone marrow and thymus, while the peripheral lymphoid organs are the structures where mature lymphocytes are activated by antigen to provoke immune responses, including lymph nodes, spleen and gut-associated lymphoid tissue (GALT). The gut microbiota is of great significance to both of these lymphoid organs, which has been confirmed by the both early and recent research.

In 1956, Miyakawa and colleagues observed undeveloped and even atrophic lymphoid tissues in germ-free guinea pigs, including defects in Peyer ‘s patches, lymph nodes, and subepithelial lymphoid tissues.^178^ Recently, Zhang et al. reported the specific mechanisms of gut-microbiota-mediated peripheral lymphatic development.^179^ They demonstrated that, driven by the commensal fungi, CD45 + CD103 + RALDH + dendritic cells (DCs) in the gut move to peripheral lymph nodes and subsequently initiate their development via retinoic acid signaling, which is marked by the lymph node cellularity increase and volume expansion.^179^ More importantly, the structural and functional maintenance of the peripheral immune organs are permanently dependent on the DCs-introduced retinoic acid signaling.^179^ Gut microbiota is also essential for the development of GALT that enhances intestinal homeostasis. For example, peptidoglycan from gut gram-negative bacteria can be recognized by the NOD1 receptor in epithelial cells, which induces the expression of downstream β-defensin 3 and CCL20, and subsequently they can activate the chemokine receptor CCR6 and induce the genesis of isolated lymphoid follicles, a kind of GALT favorable for the maintenance of intestinal homeostasis.^180^ Additionally, gut microbiota also influences intestinal homeostasis by controlling the development of thymic components. 5-OP-RU is a vitamin B2 precursor derivative produced by the gut bacteria, but not by human cells, and it can move from mucosal surface to the thymus and promote the thymic development of mucosal-associated invariant T cells, an evolutionarily conserved subpopulation of T cells that mainly exists in the mucosae, thereby enhancing mucosal homeostasis.^181^ Thus, the commensal bacteria can promote the resistance of intestinal mucosa to pathogens via immune mechanisms, and thus decrease the risk of certain types of cancer such as CRC.^182^ Moreover, lymphoid organs are the nests of immune cells as described above, thus microbiota-induced lymphatic development and maturation are important for cancer prevention.

Based on the cancer-preventing effects of the gut microbiota, concrete strains have been found to tentatively treat cancer by enhancing anticancer immunity. Recently, a consortium of 11 bacterial strains isolated from healthy human donor feces displayed capability of inducing interferon-γ-producing CD8 + T cells in the intestine, and it can enhance the efficacy of immune checkpoint inhibitor in tumor-bearing mouse models, which both imply the potential of microbes for cancer therapy.^183^

## Cancer-related microorganisms and effectors

### Typical cancer-promoting microbes

#### *Helicobacter pylori*

*H. pylori* is a gram-negative, spiral-shaped bacterium residing in or underneath the mucus layer that coats the epithelial surface of the human stomach, and it is the most important biological risk factor for gastric cancer,^184^ which has already been classified as Class I carcinogen by WHO in 1994. In China, more than 70% of non-cardia gastric cancer and more than 60% of cardia gastric cancer can be attributed to *H. pylori* infection.^185^ The stomach is the harshest environment in the human body, secreting gastric juice that contains hydrochloric acid and proteolytic enzymes, which defends against the majority of pathogenic microbes.^186^ Thus, *H. pylori* has evolved intricate mechanisms to tolerate the acidic environment for the survival and colonization in the stomach. For example, *H. pylori* produces urease, an enzyme converting urea to ammonia, and it neutralizes gastric acid and provides ammonia for bacterial protein synthesis, which contributes to the *H. pylori*-mediated gastropathy.^187^ Additionally, *H. pylori*-induced gastric carcinogenesis is mainly mediated by CagA and vacuolating cytotoxin (VacA).^188,189^ These virulence factors can be injected into the epithelial cell via the type IV secretory system,^190^ then triggering a variety of carcinogenic mechanisms that are discussed in the previous chapter. Notably, the association between *H. pylori* and an increased risk of other malignancies besides gastric cancer have also been observed, such as CRC^191^ and gastric MALT lymphoma.^192^

#### *Fusobacterium nucleatum*

*F. nucleatum* is a gram negative, anaerobic oral commensal that has long been regarded as opportunistic pathogen of periodontal disease.^193^ Recently, ample evidence has found the presence of *F. nucleatum* in colon cancer tissue,^194,195^ and it has emerged as a causal bacteria implicated in CRC.^88^ Komiya and colleagues have collected CRC and saliva samples from 14 patients, and identical *F. nucleatum* strains were detected in both CRC and saliva from 6 patients, which implies that *F. nucleatum* in CRC may originate in the oral cavity.^196^ However, *F. nucleatum* is less prevalent in the healthy gut, introducing a question about how it migrates to and colonizes the developing TME. Abed et al. injected *F. nucleatum* into the veins of tumor-bearing mice and found that it could reach the tumor tissue, concluding that *F. nucleatum* might migrate to CRC through hematogenous route.^197^ Furthermore, Fap2 surface protein, a galactose-binding lectin expressed by some *F. nucleatum* strains, could mediate fusobacterial enrichment in CRC through binding to the Gal-GalNAc, a polysaccharide overexpressed in human CRC.^197^ Another key virulence factor of *F. nucleatum* is FadA adhesin, which promote colorectal carcinogenesis through multiple mechanisms, such as triggering β-catenin signaling.^88,198^ Notably, other bacteria of *Fusobacterium* species may be involved in the development of precancerous stage of CRC, ulcerative colitis.^199^ From the clinical point of view, *F. nucleatum* in the gut may be a target for CRC prevention and therapy in the future, just like eradication of *H. pylori* for gastric cancer.

#### *Bacteroides fragilis*

*B. fragilis* is part of the normal microbiota in the human colon and has important physiological meanings, such as promoting the development of host immune system.^200^ However, ETBF, a pathogenic strain of *B. fragilis*, has been demonstrated to be correlated with tumorigenesis of colon.^201^ The key virulence factor of ETBF is an enterotoxin termed fragilysin, which is essentially a zinc-dependent metalloprotease. Chung et al. have demonstrated that fragilysin could trigger pro-carcinogenic inflammatory cascade to accelerate colon tumorigenesis.^202^ Specifically, fragilysin triggers an IL-17 immune response that selectively activates NF-κB signaling in distal colonic epithelial cells, which collectively lead to pro-tumoral myeloid cells infiltration in distal colon.^202^ Additionally, Cao and colleagues have found that ETBF could promote intestinal inflammation and CRC development by down-regulating exosomal miR149-3p secreted by CRC cells,^203^ a miRNA inhibiting tumorigenesis in other cancers.^204^

#### Epstein-Barr Virus

Besides bacteria, viruses can also promote the development of cancer, and a typical representative is Epstein-Barr Virus (EBV). EBV is one of the eight known human herpesviruses and the first cancer-associated virus, and EBV infection may lead to malignancies including lymphoma, gastric cancer and nasopharyngeal carcinoma.^205^ EBV can exert carcinogenic effect through its protein components. For example, viral protein BNRF1 can induce centrosome amplification in B-lymphocytes, which is associated with chromosomal instability, and thus increase the risk of malignant transformation.^206^ On the other hand, EBV could promote tumor immune escape in gastric cancer and nasopharyngeal carcinoma. Specifically, EBV miRNAs BART11 and BART173p could inhibit FOXP1 and PBRM1, respectively, thereby enhancing the transcription of PD-L1 that is crucial for tumor immune escape.^207^ Additionally, EBV infection could inhibit the antitumor function of NK cells infiltrated in the EBV-associated epithelial malignancies, and thus promoting the cancer development.^208^

### Cancer-inhibiting bacteria

#### *Lactobacillus*

*Lactobacillus spp*. are commonly used as food supplements, and their role in protecting against cancer was investigated initially in mice. The alleviating effects of *Lactobacillus rhamnosus, Lactobacillus acidophilus* and *Lactobacillus fermentum* on the development of colon cancer have been demonstrated in the mouse model.^209^ *L. rhamnosus* GG can stimulate type I interferon through the cGAS/STING signal transduction pathway, thereby improving the response to ICIs.^210^ Lactic acid bacteria (LAB) can effectively decrease the occurrence of CRC, which might be ascribed to the reduction of inflammatory factors. Moreover, LAB also affects the gut microbial community, which is marked by the decrease of the abundance of *Bacteroides*.^209^ Therefore, LAB is beneficial to inhibit the initiation and development of cancer.^211^ *L. reuteri* can promote the renewal and repair of intestinal epithelium and stimulate the host’s immunity.^212^ Specifically, *L. reuteri* was found to convert intraepithelial CD4 + T cells into CD4 + CD8αα + double-positive intraepithelial lymphocytes that relieve inflammatory bowel disease, thus preventing some alimentary tract cancer.^213^

##### *Bifidobacterium*

Existing evidence have found that *Bifidobacterium* species might have important cancer-inhibiting effects. For example, the tumor control effect of oral administration of *Bifidobacterium* in melanoma mice was demonstrated to be the same as that of PD-L1 antibody,^214^ and the combination of these two methods is highly effective in inhibiting tumor outgrowth.^214^ In mice fed with western style diet (WSD), an reshaped colonic microbiota composition might cause increased penetrability and reduced formation of mucus layer in the gut. However, it has been demonstrated that *Bifidobacterium longum* could regain mucus secretion in WSD-fed mice,^215^ which implies the potentially significance of *Bifidobacterium* species in the maintenance of intestinal homeostasis. β-glucan/galactan polysaccharides on the cell surface of *Bifidobacterium bifidum* were demonstrated to be crucial for the induction of Foxp3+ T regulatory cells that display suppressive capacity to experimental colitis.^216^ Thus, it is not surprsing that *B. bifidum* can regulate intestinal homestasis and prevent cancer initiation.^216^ The findings above emphasize the potential of *Bifidobacterium* in cancer treatment or prevention by affecting immune control and mucosal protection.

#### *Faecalibaculum rodentium*

*F. rodentium* and *Holdemanella biformis* (human homolog) are absent or lost in the course of tumorigenesis,^217^ both of which can produce SCFAs that control the proliferation of tumor cells and protein acetylation through the suppression of calcineurin and NFATc3 activation.^217^ Adenomatous polyposis coli (APC) gene mutations occur in more than 80% of CRCs when *F. rodentium* is applied to Apc^Min/+^ mice, or tumor growth in mice can be mitigated by treatment with azoxymethane and dextran sodium sulfate. Likewise, *H. biformis* appears to be similar to *F. rodentium* in suppressing tumor growth in the Apc^Min/+^ model by means of butyrate. Therefore, *H. biformis* may be applied in the design of cancer treatments.

#### *Streptococcus thermophiles*

*S. thermophilus* is a powerful probiotic with digestive and immune benefits, and it is normally depleted in CRC patients.^218^ More importantly, the inhibitory effect of *S. thermophilus* on tumorigenesis has been demonstrated in CRC mouse models.^218^ Specifically, oral gavage of *S. thermophilus* in CRC mosue would result in a significant reduction in tumor formation, and β-galactosidase secreted by *S. thermophile*s was found to be the active ingredient that inhibits CRC growth, which was confirmed by in vivo xenograft experiments and cell experiments^218^ In mouse CRC xenograft experiments, β-galactosidase was found to inhibit cell proliferation, cell colony formation and cell cycle arrest to promote CRC cell apoptosis, thus suppressing tumor growth.^218^ Impressively, β-galactosidase can increase the richness of another two probiotics, *Lactobacillus* and *Bifidobacterium*, suggesting a synergistic effect.^218^ *S. thermophilus* can also affect tumor growth by releasing folate,^219^ a major dietary element that plays an important role in cell metabolism and DNA replication, repair, methylation, and nucleotide synthesis. Research suggests that folate deficiency is fairly prevalent in humans,^220^ and the folate released by *S. thermophilus* might be involved in tumor suppression. In addition, *S. thermophilus* has an effect on the lymphocyte profile, the severity of colitis, and the regulatory T-cell response.^221^

### Cooperative and competitive relationship among microbes in cancer development

Symbiotic, antagonistic and neutral relationships among the gut microbes exist, the former two of which may be involved in the carcinogenic mechanisms of microbes. It is well known that *F. nucleatum* is an oral-derived bacteria closely associated with the occurrence and progression of CRC.^38,196^ Thus, if *F. nucleatum* grows well in the oral cavity, it may be beneficial to its migration to CRC. Sakanaka et al. have discovered cooperative relationship between *F. nucleatum* and *Streptococcus gordonii*, another symbiotic bacteria colonized on the surface of human oral mucosa.^222^ Specifically, *S. gordonii* could secret ornithine, which in turn support the growth and biofilm development of *F. nucleatum* in oral cavity.^222^ Although the direct impact of this cooperation on the development and progression of CRC has not been confirmed, it is likely to promote the colonization of *F. nucleatum* in the cancer foci by enhancing its viability, which is linked to the development of CRC.

Additionally, carcinogenic microbes can be antagonized by some probiotic. For example, *B. bifidum* strain BF-1 can suppress the expression of *Hp*-induced genes in human cells, most of which are related to the NF-κB signaling pathways.^223^ Because *Hp*-induced NF-κB signaling can promote the malignant transformation via regulating chronic inflammation,^224^ BF-1 can protect host cells from carcinogenesis.

### Effects of metabolites in cancer development

#### SCFAs/DCA

SCFAs, including propionic acid, butyrate and tryptophan, play a key role in a variety of host biochemical and physiological functions, e.g., maintaining intestinal barrier integrity and intestinal motility, as well as regulating immunological function and the gut-brain axis.^225,226^ Butyrate is one of the most widely studied SCFAs, which is produced through fermentation of dietary fibers by gut microbes. It can not only serve as an energy source for normal colonocytes, but also reduce the risk of CRC.^227^ It is well known that the destruction of intestinal mucosal barrier acts as an accomplice in the occurrence of CRC, while butyrate can repair and enhance the function of the gut barrier.^228^ Peng et al. demonstrated that one of the specific mechanisms by which butyrate enhances the intestinal barrier is to promote the assembly of tight junctions through activating AMP-Activated Protein Kinase.^229^ Additionally, butyrate also stimulates the expression of MUC2 in intestinal epithelial cells, which can enhance the mucous layer involved in the formation of intestinal barrier.^230^ On the other hand, butyrate can directly inhibit the proliferation of CRC cells by remodeling metabolism, which is manifested by the inhibition of Warburg effect and the enhancement of energy metabolism.^231^ Notably, β-hydroxybutyrate, a chemical substance structurally similar to the butyrate, also suppresses CRC by inducing the transcriptional regulator Hopx through the surface receptor Hcar2.^232^ What’s more, butyrate also inhibits other types of cancer. For example, sodium butyrate combined with cisplatin can enhance the apoptosis of gastric cancer cells through the mitochondrial apoptosis-related pathway, which might be an underlying strategy for gastric cancer.^233^

DCA, a secondary bile acid produced by gut microbes from primary bile acids through 7α-dehydroxylation, has an extensive range of effects on host metabolism and plays an important role in health.^234^ However, DCA may also play a key role in cancer development by mediating a variety of signaling pathways, including EGFR-MAPK and β-catenin signaling, and the p53 pathway.^235^ For example, obesity-induced increased DCA can provoke senescence-associated secretory phenotype in hepatic stellate cells through enterohepatic circulation, which in turn promotes the secretion of tumor-promoting factors and inflammatory factors associated with hepatocellular carcinoma.^77^ Furthermore, DCA also promotes CRC progression through antagonizing intestinal farnesoid X receptor.^236^ Besides, bioactive molecules generated from gut-bacteria-mediated bile acid metabolism may determine immune cells differentiation, which is crucial for tumor immunology.^237^ Taurine-conjugated bile acids, another metabolite of intestinal microbes, can produce hydrogen sulfide and DCA and promote the growth of CRC tumors.^235^ In addition, the bile acid pool in colon may influence FOXP3 + Treg cells, indicating a crucial role in immunity regulation.^238^ Interestingly, DCA may serve as a tumor suppressive factor in gallbladder cancer, which suggests an underlying strategy for the malignancy.^239^

#### Tryptophan and trimethylamine N-oxide (TMAO)

Tryptophan (Trp) is an essential amino acid that can be metabolized through the kynurenine pathway and microbial transformation, both of which are significant for host health. However, the two metabolic pathways of Trp are different in colon carcinogenesis, which may allow the immune escape of tumor cells. Colon cancer cells are more likely to absorb and process tryptophan than normal colonic epithelial cells.^240^ Specifically, the oncogene c-Myc can promote Trp absorption by upregulating Trp transporters SLC7A5 and SLC1A5 and accelerate Trp metabolism through increasing the level of related enzymes in the cytoplasm in colon cancer cells, both of which contribute to T cell inactivation and protein synthesis in the process of carcinogenesis.^225^ In addition, kynurenine, the intermediate product of Trp metabolism, can accelerate the progression of pancreatic cancer.^241^ Kynurenine also regulates immunity by promoting the nuclear translocation of AhR, which is an inflammatory and immune-related transcription factor.^242^ Moreover, *Lactobacilli* can convert tryptophan into indole-3-aldehyde that acts as an AhR agonist by increasing the expression of IL-22 and enhancing the activity of Th17 cells.^243^

TMAO is metabolized in the liver from trimethylamine (TMA) synthesized by host gut microbes,^244^ and it has been demonstrated to increase the risk of cardiovascular disease such as myocardial infarction and stroke.^245,246^ Omnivorous humans produce more TMAO than vegetarians,^247^ and its level is associated with the risk of cancer, including CRC.^248^ Furthermore, TMAO is capable of activating the PERK-mediated response and thus activating forkhead box protein O 1, which is crucial for metabolic regulation.^244^ Accordingly, the formation of TMA and TMAO can be the connections among diet, the gut microbiota and cancer. Gaining a better understanding of the role of TMAO in the pathogenesis of cancer will be favorable for cancer prevention and control.

#### Insulin resistance and inosine

In a Swedish study, the microbiota community of the pancreas in patients with impaired glucose tolerance (IGT) or type 2 diabetes mellitus (T2DM) was found to be altered.^249^ It is interesting to note that the abundance of butyrate-producing bacteria decreased in both prediabetic and T2DM patients.^249,250^ Insulin resistance has been found to be strongly related to microbial dysfunction.^249^ In a further study, gut bacteria associated with T2DM was found to impaire glucose tolerance and insulin signaling by producing a metabolite termed imidazole propionate from histidine,^251^ thus a relatively high concentration of imidazole propionate can be detected in T2DM patients.^252,253^ Insulin resistance has the potential to stimulate the growth of cancer via mTOR activation,^253,254^ partly because of imidazole propionate,^251^ and it leads to metabolic changes that promote cancer growth.^249,255,256^ Thus, it is possible that gut microbiota dysfunction that induces insulin resistance may contribute to tumor development.

Three bacteria, *Bifidobacterium pseudolongum, Olsenella*, and *Lactobacillus johnsonii*, have been shown to exert a positive effect on the effectiveness of immunosuppressors in mouse models due to the metabolite inosine.^257^. In fact, inosine is an immunotherapy-promoting metabolite and has been experimentally shown to have an effect on colon cancer, bladder cancer and melanoma.^257^ Mechanistically, inosine triggers the activation of Th1 cells by regulating T-cell-specific A_2A_R signaling. Thus, the development of inosine-based adjuvant therapies may enhance the efficacy of ICIs. In the future, a better understanding of the underlying mechanisms of inosine will be of great help to formulate proper ICI-based therapy strategies.

#### Niacin and vitamin B

Niacin acts as the precursor of nicotinamide dinucleotide (NAD) and NAD phosphate (NADP), both of which are involved in redox reactions. NAD also correlates transcriptional regulation with cellular energetics.^258^ Monosaccharides are produced in the fermentation of carbohydrates, which are further catabolized to produce pyruvate and NADH molecule.^225^ G protein-coupled receptor 109 A (GPR109A) acts as a receptor for both niacin and butyrate,^259^ through which niacin can inhibit the growth of colon cancer.^260^ Niacin also displays beneficial effects on colitis by prostaglandin D2 enhancement.^261^ Vitamin B contributes largely to the synthesis of DNA and protein, and it also plays a key role in the metabolism of ser-gly one-carbon.^225^ Gut bacteria can synthesize a group of B vitamins, including B1, B2, B3, B5, B6, B7, B9 and B12, which are essential for human health.^225^ More importantly, B vitamins will impact tumorigenesis through the SGOC pathway.^262,263^

#### Diacetyl spermine/oncotoxins

Bacterial biofilm, which contributes to the polyamine pool, plays a nonnegligible role in changing the TME.^264^ Polyamine metabolites are upregulated in the tissues of cancer patients.^265,266^ It is important that antibiotic therapy can clear the bacterial membrane, thus reducing the number of N(1),N(12)-diacetylspermine^267^ and polyamine metabolites that promote the growth cancer. Mechanistically, polyamine is associated with the proliferation of eukaryotes. Bacteria in *Eggerthellaceae* family have been found to produce urolithin,^268^ which is derived from polyphenols in some fruits with anti-inflammatory and antioxidative capabilities, and activate AhR to upregulate tight junction proteins,^219^ thus having antitumor activity. Moreover, the carcinogenic versions of the bacteria *E.* *coli* and *B. fragilis* may produce oncotoxins that accelerate carcinogenesis.^12^ Specifically, cytolethal-derived toxin from enteric pathogens (*Escherichia and Bacillus spp*.) and colibactin from *Enterobacteriaceae* are demonstrated to be tumorigenic due to their DNA damage effects.^269^ Thus, the modification of microbes and their products may be beneficial in the treatment of cancer since oncogenic toxins and metabolites produced by microbes can contribute to carcinogenesis.^270^

### Targeting the gut microbiota in clinical cancer treatment

**Fig. 3:**
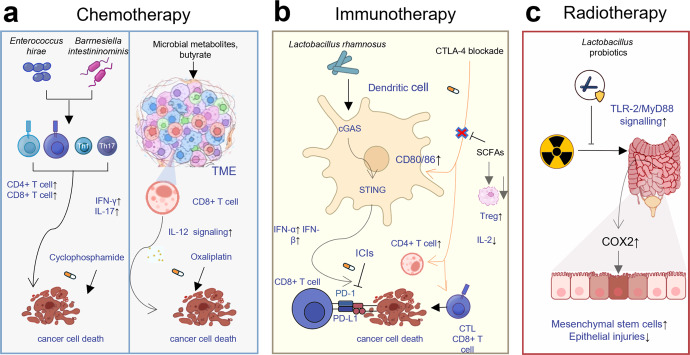
The mechanisms of microbiota impacting efficacy of cancer treatment. **a** Specifically, administration of *Enterococcus* and *Barnesiella* can restore the antitumor efficacy of cyclophosphamide-based chemotherapy through stimulating tumor-specific T cells and producing IFN-γ, and butyrate, a product of dietary fiber fermented by gut microbes, can increase the anticancer effects of oxaliplatin-based chemotherapy by regulating the function of CD8 + T cells in the TME through IL-12 signaling; **b** *Lactobacillus rhamnosus* was illustrated to stimulate the antitumor activity of PD-1 immunotherapy through cGAS-STING signal pathway, activating IFN-α, β signaling, and activating cytotoxic CD8 + T cells; SCFAs limit the antitumor effects of CTLA-4 blockade via alleviating Treg cells, and higher concentration of butyrate could decrease the anticancer activity of Ipilimumab by inhibiting the accumulation of related CD4 + T cells; **c** probiotics can protect gut mucosa from radiation injury through a TLR-2/COX-2-dependent manner, stimulating mesenchymal stem cells to the crypt. (FMT fecal microbiome transplantation, SCFAs short-chain fatty acids, IL interleukin, IFN- γ interferon γ, CTLA-4 cytotoxic T lymphocyte-associated antigen 4, Treg cell regulatory T cell, TLR Toll-like receptor, COX-2 cyclo-oxygenase-2)

The gut microbiota can be regarded as a special organ, and its composition can be adjusted in various ways. More importantly, with the in-depth study on gut microbes in recent years, researchers have found a strong relationship between gut microorganisms and anticancer treatment efficacy,^271–273^ providing us with a new anticancer direction,^274,275^ which is to enhance efficacy and reduce therapeutic toxicity of conventional anti-cancer therapies by modulating the microbial composition in the gut,^276^ although we are still far from a full-fledged microbial anticancer treatment.^277–279^ The relationships between gut microbes and anti-cancer treatment and the current as well as emerging microbial interventions for cancer therapy are summarized here (Fig. 3).

### Gut microbiota and cancer therapy efficacy

#### An emerging role: The microbiota affecting immunotherapy

Cancer immunotherapy, as one of the revolutionary advances in the last ten years, mainly includes immune checkpoint therapy, typified by cytotoxic CTLA-4 and PD1, and adoptive T-cell therapy (ACT), represented by chimeric antigen receptor T-cell (CAR-T) therapy as well as cancer vaccines,^280^ and it has occupied an increasingly important position in the comprehensive treatment of cancer.^281–292^ To date, immunotherapy has improved treatment outcomes for many cancer patients, but there are still a large proportion of patients who receive immunotherapy with little benefit, showing primary or acquired resistance to treatment,^293^ including patients with melanoma,^294^ and patients with non-small cell lung cancer.^295–298^ Available evidence suggests that this discrepancy in efficacy may be linked with gut microbes.^48,299–304^

Among all immunotherapies, immune checkpoint inhibitors (ICIs) therapy is the most mature, and its main mechanism is upregulating the immune killing effect of T cells by targeting coinhibitory molecules including PD-1/ PD-L1, to boost the endogenous host immunity and prevent tumor cells from immune escape.^305^ Indeed, the response to ICIs seems to be predictable based on the gut microbial composition,^47,306,307^ because gut microbes can also be involved in the adjustment of host immunity, which may in turn indirectly affect the response of cancer patients to ICIs.^47,306,308–312^ For instance, active *enterococci* secretes SagA, an ortholog of the NlpC/p60 peptidoglycan hydrolase catalyzing the production of immune-active muropeptides, that can bind to NOD2, a key pattern recognition receptor, through which host immunity can be enhanced via multiple pathways and thus may ultimately augment anti–PD-L1 antitumor efficacy.^313^

*L. rhamnosus* was illustrated to stimulate the antitumor activity of PD-1 immunotherapy by triggering dendritic cells to produce IFN-α and IFN-β through the cGAS-STING signaling pathway.^210^ *Bifidobacterium* plays antitumor roles by inducing the maturation of dendritic cells, activating IFN-α and IFN-β signaling, and stimulating cytotoxic CD8 + T cells.^214^ In addition, vitamin B5, produced by intestinal bacteria and contained in most food, could promote the generation of IL-22-producing Tc22 cells, a kind of immune cell that has particularly efficient antitumor effects and correlates with stronger immunotherapy responses.^314^ As per these findings, novel therapeutic strategies based on microorganisms have been developed to modulate the gut microbiota to improve the clinical response to ICIs^66,67,315^ and to reduce therapeutic toxicity.^316,317^

However, due to the diversity within the gut microbiota, there are bound to be microbes that have the exact opposite effects on ICIs. For example, SCFAs limit the antitumor effects of CTLA-4 blockade,^318^ and high concentration of butyrate in cancer patients could decrease the anticancer activity of ipilimumab by inhibiting the accumulation of related T cells and IL-2 impregnation.^318^ It is well known that a large amount of the SCFAs in the human body comes from the fermentation of dietary fiber by intestinal bacteria. Therefore, some intestinal bacteria are actually able to indirectly inhibit the antitumor effect of CTLA-4 blockade by producing corresponding metabolites. From the findings of these studies, it is not difficult to understand the presence of reticular relationships among gut microbes, diet, human immunity and immune checkpoint inhibitors. First, diet and gut microbiota have effects on each other.^319,320^ Specifically, diet can influence the composition of gut microbiota, and gut microbes can participate in the digestion and absorption of nutrients. Secondly, healthy diet and balance gut microbiota are both essential for the maintenance of human immunity,^321,322^ which in turn defense against the invasion of pathogenic microbes and balance the gut microbiota.^323^ More importantly, as discussed above, gut microbes participate in the metabolism of nutrients, producing metabolites that affect the body’s immunity, ultimately impacting the effects of ICIs.

In addition to affecting the efficacy of ICIs, gut microbes also have an impact on ACT.^324^ To date, little research regarding the influence of gut microbes on ACT has been conducted, but such effect does exist.^325^ Depleting the gut microbes in tumor-bearing mice undergoing ACT with vancomycin slowed their tumor growth, whereas neomycin and metronidazole had no similar effect.^326^ Notably, relevant observational studies were conducted to confirm the impact of gut microbes on ACT. Smith M et al. retrospectively collected and analyzed clinical data from patients with acute lymphoblastic leukemia and patients with non-Hodgkin lymphoma, and they found that exposure to antibiotics, e.g. meropenem, piperacillin/tazobactam and imipenem/cilastatin, during the 4 weeks before CAR-T-cell therapy was associated with worse clinical outcomes and prognosis; furthermore, they found a higher abundance and richness of *Ruminococcus, Bacteroides* and *Faecalibacterium* in stool samples were correlated with a better response to CD19 CAR-T-cell therapy.^327^ Therefore, similar to the effects on immune checkpoint therapy, distinct gut microbial compositions do affect the efficacy of ACT, and the mechanisms behind these effects need to be further investigated.

#### Chemotherapy efficacy is closely linked to the gut microbial composition

Chemotherapy is one of the major treatments for cancer, but not all patients respond well to it. Taking patients with stage II and III gastric cancer as an example, postoperative adjuvant chemotherapy can significantly improve the five-year survival rate of this population, whereas there are still a considerable proportion of patients who do not benefit from chemotherapy.^328^ One reason for cancer patients respond differently to the identical chemotherapy drugs may be the differences in the composition of the gut microbiota among individuals. In other words, some microbes in the gut are involved in regulating the efficacy of chemotherapy,^329,330^ and this regulation includes both promoting and inhibitory effects.^44,331–333^

Gemcitabine is a commonly used chemotherapy agent for pancreatic ductal adenocarcinoma (PDAC). Gut microbes are involved in the pharmacokinetics of chemotherapy drugs, and the efficacy of gemcitabine for PDAC may be influenced by intestinal microorganisms.^334^ For instance, *Gammaproteobacteria* is able to metabolize gemcitabine and convert it to the inactive form, 2′,2′-difluorodeoxyuracil.^43^ Therefore, in the future, it may be possible to increase the anti-cancer effect of gemcitabine by combining antibiotics against *Gammaproteobacteria* with chemotherapy. In addition to the negative effects, however, a gut microbial metabolite, butyrate, can enhance the efficacy of gemcitabine against cancer cells by inducing apoptosis.^335^

Cyclophosphamide, another widely used immunostimulatory agent for chemotherapy, has been demonstrated to have mitigated antitumor efficacy in antibiotic-treated or germ-free mice due to a lack of Th1- and Th17- related immune responses. Interestingly, the administration of *Enterococcus* and *Barnesiella* can restore its antitumor efficacy through the stimulation of tumor-specific CD8 + and CD4 + T cells and Th1 and Th17 cells. In addition, erlotinib is a highly specific tyrosine kinase inhibitor that can reversibly inhibit epidermal growth factor receptor mutations and is mainly used for targeted therapy after the failure of chemotherapy for non-small cell lung cancer (NSCLC). Recently, gut microbes were found to be positively correlated with erlotinib treatment outcomes.^336^ Specifically, *Bacteroides xylanisolvens and Bacteroides ovatus* were positively correlated with the treatment outcomes of erlotinib, and oral administration of the bacteria could significantly enhance the efficacy of erlotinib and induce the expression of C-X-C motif ligand 9 (CXCL9) and IFN-γ in a murine lung cancer model.^336^ More importantly, the microbial signature that enhances the efficacy of erlotinib may also be used in the treatment of other cancers, as this signature is independent of cancer type.

The efficacy of oxaliplatin varies individually and it may be related to the presence of certain metabolites of gut microbes. For example, butyrate, a product of dietary fiber fermented by gut microbes, could increase the anticancer effects of oxaliplatin by regulating the function of CD8 + T cells in the TME through IL-12 signaling.^337^ Therefore, selected gut microbial metabolites may be used as adjuncts to oxaliplatin to enhance anticancer responses in the future. In addition, commensal microbes can also influence the cancer response to oxaliplatin by modulating the functions of myeloid-derived cells within the TME.^338^ Through related studies using cancer mouse models, it was found that tumor-infiltrating myeloid-derived cells responded poorly to cancer treatment under antibiotic treatment or sterile conditions, resulting in insufficient production of reactive oxygen species and cytotoxicity following chemotherapy, which ultimately led to a decline in the efficacy of oxaliplatin.^338^ Therefore, gut microbiota dysbiosis in cancer patients may be one of the reasons for resistance to chemotherapy drugs, and interventions targeting the gut microbiota could be a promising strategy to improve cancer chemotherapy efficacy.

#### Bidirectional relationships between the gut microbiota and radiotherapy

Radiation therapy (RT) is a long-established cancer therapy that has been used to treat most types of cancer for more than one hundred years. The basic principles of radiotherapy include two aspects: on the one hand, the DNA of cancer cells is destroyed by ionizing radiation directly to kill cancer cells; on the other hand, RT indirectly kills cancer cells by causing reactive oxygen species-dependent damage to DNA.^339^ However, RT not only kills cancer cells but also can cause varying degrees of adverse effects on normal tissues and commensal microorganisms in the body, especially those in the gut.^339,340^

A bidirectional relationship between RT and the gut microbiota exists.^341^ One of the adverse events of radiotherapy is gut microbiota dysbiosis, which is typically characterized by a relative decrease in the richness of favorable microbes, e.g., *Bifidobacterium*, and an increase in the relative richness of harmful microorganisms such as *Fusobacteria and Proteobacteria*,^342^ and these changes in the composition of gut microbiota in turn exacerbates radiation-related complications, such as radiation enteropathy.^343,344^ However, the presence of some commensal microbes is critical for improving the efficacy of radiotherapy and moderating RT-related adverse events.^340^ Working on mouse models of breast cancer, Shiao et al. found that depleting the gut bacteria with an antibiotic cocktail of ampicillin, imipenem, cilastatin, and vancomycin before radiotherapy resulted in faster tumor growth and shorter survival of tumor-bearing mice than RT alone, and a similar situation has been observed in mouse models of melanoma.^345^ However, what is thought-provoking is that when the tumor-bearing mice were administered the abovementioned antibiotic cocktail alone without receiving radiotherapy, their tumors grew more slowly than control group, which was somewhat paradoxical to previous experiments.^345^ In this regard, the depletion of gut microbes might cause changes in metabolism, which ultimately leads to a reduction in tumor-promoting metabolites such as serum glucose and SCFAs.^345^ In addition to bacteria, fungi have also been found to be associated with the response to radiation therapy, which suggests that other types of secondary microbes in the gut microbiota can also influence the efficacy of radiotherapy, although this effect may be negative.^345^ Accordingly, the relationship among gut microbes, tumors and radiotherapy is reticular, with a very broad research space.

#### Gut microbiota and therapy-related side effects

Conventional anticancer therapies have their own side effects, and even immunotherapy, which has been very popular in recent years, is no exception.^346,347^ It has been noted that the gut microbiome is associated with the toxicity of traditional anticancer therapies and that modulating the components of the gut microbiome may alleviate related toxicity.^348^ Therefore, understanding the relationship between different microbes and the side effects of traditional anticancer therapy is particularly important for individualized mitigation of these adverse events.

Immune checkpoint therapy can cause severe inflammatory side effects, and one of its most serious adverse events is colitis.^349^ Some researchers have found that in patients with severe ICI-related colitis, the abundance of *Lactobacillus* in the gut decreased obviously, and subsequent studies confirmed that the ICI-related colitis could be moderated via oral administration of this probiotic.^350^ Mechanistically, the ability of *L. reuteri* to inhibit ICI-related colitis is associated with a decrease in the distribution of group 3 innate lymphocytes.^350^ Moreover, the profiling of the gut microbiota in melanoma patients receiving combined PD-1 and CTLA-4 blockade suggested that a higher abundance of *B. intestinalis* was related to adverse events of immunotherapy.^317^ Similarly, the immunotoxicity of a novel immunotherapy drug, immune agonist antibodies (IAAs) targeting costimulatory molecules, is also linked to the gut microbiota.^351^ Specifically, germ-free and antibiotic-treated mice had fewer complications following treatment with the IAAs anti-CD40 and anti-CD137 than normal or microbial recolonized germ-free mice.^351^ Taken together, these findings indicate that gut microbiota signatures are clearly associated with the toxicity of ICIs, and the association could be a new breakthrough point for developing new therapeutic strategies in the future.

Chemotherapy, while saving cancer patients, also has many side effects, including intestinal flora imbalance, mucositis and diarrhea.^352^ For example, irinotecan, a common chemotherapy drug, can kill cancer cells, but it can also cause the death of normal epithelial cells and commensal microorganisms in the gut. Therefore, irinotecan tends to cause gastrointestinal toxic side effects and intestinal flora imbalance. The β-glucuronidase secreted by gut bacteria can prolong the clearance time of irinotecan in vivo, so gut microbes can exacerbate irinotecan-induced gastrointestinal toxicity.^353^ In addition, irinotecan-induced intestinal dysbiosis can exacerbate the gastrointestinal toxicity of irinotecan by damaging the intestinal mucosal barrier.^354^ Therefore, gut microbes are clearly involved in the mechanisms of irinotecan-related adverse events. Direct inhibition of β-glucuronidase may be able to reduce the gastrointestinal toxicity of irinotecan and improve patients’ quality of life.^355^ Moreover, the side effects of irinotecan can also be alleviated by taking certain probiotics, e.g. *E.* *coli* strain Nissle 1917, which could regulate gut barrier epithelial function, alleviate gut dysbiosis, and ultimately reduce intestinal complications caused by irinotecan.

RT not only kills cancer cell, but also causes varying degrees of adverse effects on normal tissues and disrupts the diversity and abundance of commensal gut microorganisms.^340,356^ The RT-induced dysbiotic gut microbiome in turn exacerbates the gastrointestinal toxicity of radiotherapy.^340^ Conversely, certain probiotics or probiotic preparations, such as *L. rhamnosus* and VSL#3 (a probiotic preparation composed of *Bifidobacterium species, Lactobacillus*, and *Streptococcus*) could protect the intestinal epithelium from injury and reduce the side effects of RT.^357–359^

Cancer surgery, especially surgical resection of gastrointestinal cancer with alimentary reconstruction, has many postoperative complications, the most common of which are surgical site infections and anastomotic leaks. Despite improvements in preoperative preparation, surgical techniques, and postoperative care over the years, anastomotic leaks and postoperative infections occasionally occur with serious consequences, including acute peritonitis and even death. To reduce the risk of these two complications after surgery, patients typically undergo preoperative bowel preparation to empty their colon of stool and take antibiotics to prevent infection. Clearly, these measures reduce the risk of postoperative complications by reducing the abundance of the relevant microbes in the patient’s gut. Metabolism following gastrectomy is related to microbial function alterations, such as the biosynthesis of organic compounds and nutrient transport.^360^ Related studies have confirmed that intestinal microbiota composition could predict short-term prognosis after gastrointestinal cancer surgery.^361–363^ For instance, low microbial diversity and mucin-degrading members of the *Bacteroidaceae* and *Lachnospiraceae* families are associated with postoperative anastomotic leaks.^364,365^ Surgical site infections are caused by various factors, such as drug-resistant and virulent microorganisms that have emerged as a result of globalization, antibiotic exposure, and the application of prolonged and invasive treatments.^366^ Curiously, is there some kind of commensal microorganism that can promote anastomosis healing and prevent surgical site infections? There is literature suggesting that some probiotics may be able to inhibit pathogenic microorganisms associated with postoperative infections. For example, some strains in *Lactobacillus* and *Bifidobacterium* are capable of inhibiting the growth of clinically isolated methicillin-resistant *Staphylococcus aureus*, a multidrug resistant microorganism that is a major nosocomial pathogen and relates to postoperative infections, by direct cell competitive exclusion as well as the production of inhibitors.^367^ In the future, it may not be impossible to develop microbial therapies to improve postoperative prognosis by targeting these related microorganisms.

### Current and emerging microbial interventions/therapeutic strategies for cancer therapy

With a more comprehensive and in-depth understanding of the gut microbiome gained in recent years, an increasing number of potential microbial interventions for cancer therapy have been proposed,^368^ including FMT, treatment with prebiotics, probiotics or antibiotics, and dietary interventions, which have already illustrated great prospect of microbial therapies. In the future, some microbial strategies mentioned above may be translated to widely-accepted anti-cancer interventions.

#### Fecal microbial transplantation (FMT) applications in clinical medicine

FMT refers to the transplantation of the functional flora of healthy donors into the intestinal tract of recipients for the purpose of treating diseases, and it was first used to treat severe diarrhea more than 1600 years ago during the Eastern Jin Dynasty in ancient China, when Chinese doctors issued a prescription called “Huanglong soup”.^369^ The recipients mentioned above can not only refer to patients with diarrhea, but cancer patients. The transplantation of fecal microbes from patients with a complete response to ICIs into immunotherapy-refractory melanoma patients could reduce the tolerance to ICIs.^66,67^ Following FMT, the abundance of favorable microorganisms, including *Ruminococcus* and *Bifidobacteriaceae*, in the recipient’s gut significantly increased, which is associated with improved clinical responses.^66,67^ Mechanisitically, melanoma patients with an improved response to ICIs following FMT showed enhanced intratumoral and intestinal immune infiltration.^66,67^ The above changes all indicate that FMT can improve the anticancer efficacy of ICIs by modifying the recipient’s gut microbial composition and then improving host immunity. In addition to improving the efficacy of anticancer therapy, available evidence has confirmed that FMT could cure adverse events occuring during cancer treatment. A recent case series reported that the ICI-induced colitis of two patients was cured by FMT, accompanied by a remodeling of the gut microbiome.^370^ In contrast, corticosteroids that are thought to play a role in anti-integrin and anti-TNF before FMT were also administered to both patients, but none of them worked.^370^ In addition, some researchers conducted a randomized controlled trial in tyrosine kinase inhibitor (TKI)-treated patients with renal cell carcinoma and found that the transplantation of fecal microbes from healthy donors into patients was able to cure TKI-induced diarrhea.^371^ These study findings all illustrate the broad application prospects of FMT in cancer treatment in the future.

#### Defined microbial consortia and probiotics

One of the disadvantages of FMT is the nonspecific entire microbiome of healthy donors or responders is inserted into the recipient’s gut. To more specifically modulate the recipient’s gut microbiota to more efficiently improve their response to other forms of cancer treatment, it has been envisaged to more purposefully combine one or several microorganisms into a single formulation.^372^ Existing evidence has demonstrated that certain microbial consortia could indeed boost the efficacy of conventional cancer therapies. In an RCT evaluating metastatic renal cell carcinoma, patients treated with both ICIs and CBM588, a bifidogenic live bacterial product, were found to have significantly longer progression-free survival and higher response rates to ICIs than patients treated with ICIs alone,^70^ demonstrating that bifidogenic live bacterial products may be able to promote the anticancer effects of ICIs on metastatic renal cell carcinoma. Moreover, a consortium composed of 11 bacterial strains from the feces of healthy donors was isolated to stimulate CD8 + T cells that reduce IFN-γ without causing inflammation.^183^ Furthermore, subsequent studies confirmed that the transplantation of this bacterial consortium into mice could enhance the therapeutic effect of ICIs.^183^ FMT may abrogate ICI-associated colitis by increasing the number of regulatory T cells within the gut mucosa.^370^ Although the great potential of microbial consortia as an adjuvant therapy for cancer has been demonstrated in these studies, many problems are still encountered, including the route of administration, dosage and cross-infection.

In addition to the above-mentioned bacterial consortia, the auxiliary role of probiotics in cancer treatment has also attracted widespread attention. For example, a probiotic compound consisting of four strains could significantly improve the prognosis of gastric cancer patients receiving gastrectomy, which was reflected in the reduction in the postoperative inflammation risk, the enhancement of immunity, the restoration of gut microbial homeostasis and the promotion of postoperative recovery.^68^ In addition, cancer patients who receive other forms of anticancer treatment may improve their prognosis and alleviate adverse events by taking probiotics.^373,374^ Unfortunately, probiotics can also have negative effects on cancer treatment. The administration of commercially available probiotics for melanoma patients was found to be associated with worse response to ICIs,^315^ which reminds us that the role of microorganisms is very complex and requires continuous in-depth exploration.

#### Targeted antibiotics

Long-term use of broad-spectrum antibiotics may lead to gut dysbiosis, which is often associated with poor clinical outcomes of cancer patients.^306,375,376^ However, relevant studies have also demonstrated that carefully selected ATB regimens could indirectly exert anticancer effects and reduce complications during cancer treatment by targeting oncogenic or pathogenic microorganisms.^377–379^ It is commendable that carefully selected antibiotics targeting carcinogenic organisms can be used not only to improve the clinical outcomes of cancer patients but also to help prevent cancer in populations at high risk of cancer or those with precancerous lesions. Dietary heme, a metabolite of red meat, could induce the cytotoxicity of colonic contents, which in turn promotes compensatory hyperproliferation and hyperplasia of the epithelium, ultimately leading to an increased risk of colon cancer, while antibiotics such as ampicillin, metronidazole, and neomycin could strengthen the mucus barrier and epithelial integrity by killing mucin-degrading bacteria and sulfur-producing bacteria, thereby preventing heme-dependent cytotoxic micelles from reaching the gut epithelium and ultimately reducing the risk of colon cancer caused by heme.^380^

#### Bacteriophage-based strategies

Some scholars have also proposed the idea of using phages to modulate the composition of the gut microbiome for anticancer purposes. Some researchers have covalently linked azide-modified phages with irinotecan-loaded dextran nanoparticles to inhibit *F. nucleatum* playing an unignorable role in the tumorigenesis of CRC, and it was confirmed that the administration of the joint unit could significantly enhance the efficacy of chemotherapy drugs for CRC.^381^ Moreover, bacteriophages can also remodel the TME. The M13 phage could specifically bind to *F. nucleatum*, and researchers assembled silver nanoparticles (AgNPs) on the surface capsid protein of this phage (M13@Ag).^382^ Subsequently, it was confirmed that M13@Ag could eliminate *F. nucleatum* in the gut, reduce the amplification of immunosuppressive myeloid-derived suppressor cells caused by *F. nucleatum* in tumor sites, and then remodel the TME against CRC.^382^ In addition, M13 phage could also activate antigen-presenting cells to further awaken the body’s immune system for CRC suppression.^382^

#### Genetically engineered/surface-modified bacteria strategies

In addition to indirectly improving the efficacy of anticancer therapy by modulating the composition of gut microbes, genetic engineering and surface modification have been used to modify bacteria for direct anticancer purposes in recent years.^383^ Some researchers have successfully enhanced the anticancer activity of violacein under hypoxia by transferring the violacein biosynthetic cluster into the oncolytic strain VNP20009 of *Salmonella*, which acts as a targeted delivery vehicle with tumor-colonizing properties.^384^ Another approach is surface modification, that is, making various modifications to the envelope structure of bacteria to endow them with new biological properties.^385^ Li et al. decorated the surface of bacteria with checkpoint-blocking antibodies and tumor-specific antigens, and the modified bacteria achieved effective antitumor efficacy in antigen-overexpressing tumor models.^386^

#### Diet and prebiotic strategies

Dietary intervention is an indirect and more moderate strategy than the aforementioned approaches that directly modulate gut microbial composition. Many preclinical studies have confirmed that dietary intervention can alleviate chemotherapy-induced toxicity^387^ and enhance the body’s anticancer immune surveillance during immunotherapy.^315,388–391^ Relevant dietary strategies include fasting-mimicking diets(FMDs),^392^ high-fiber diets,^315,393^ and ketogenic diets.^388^ FMDs such as cyclic fasting, low-carbohydrate diets and calorie restriction could reshape anticancer immunity in cancer patients by enriching IFN-γ, enhancing intratumoral Th1/cytotoxic responses, and inducing the contraction of regulatory T-cell compartments, and stimulating other immune signatures related to favorable clinical outcomes.^394^

The concept of prebiotics was first proposed in 1995 by Glenn R Gibson, and it refers to indigestible food components that influence the host by selectively promoting targeted bacterial species growth in the colon to improve host health.^395^ In recent years, with the deepening of research, some prebiotics have been found to shift the metabolism of the gut microbiota in a direction that is beneficial to anticancer treatment and improve the efficacy of anticancer treatment.^396,397^ Prebiotic inulin can exert its effects through a variety of pathways, thereby increasing its activity in the intestine and enhancing the function of gut T lymphocytes, thereby overcoming resistance to MEK inhibitors.^398^ Ginseng polysaccharides, a prebiotic derived from ginseng, could enhance the cancer response to PD1 inhibitors by decreasing the ratio of kynurenine/tryptophan and increasing the microbial metabolite valeric acid, thereby contributing to the induction of Teff cells and the suppression of regulatory T cells.^399^ In addition, Ganoderma lucidum polysaccharide could alleviate AOM/DSS-induced gut dysbiosis, increase the production of SCFAs, and alleviate endotoxemia by suppressing the TLR4/MyD88/NF-κB pathway, which may be associated with its ability to reduce AOM/DSS-induced colitis and tumorigenesis.^400^ These two findings remind us that we can continue to extract new anticancer prebiotics from traditional Chinese herbal medicine to develop more cancer treatment options in the future.

#### Nanotechnology modulation of the gut microbiota for cancer therapy

Over the past decades, nanotechnology has been studied for cancer treatment, but there is little research on how to modulate the gut microbiota with nanotechnology to indirectly achieve anticancer goals. Recently, some researchers have used the membrane of *Helicobacter pylori* to fabricate a bacterial outer membrane-coated nanoparticle, which could compete with *H. pylori* to inhibit pathogen adhesion.^401^ Although the study did not definitively demonstrate that the inhibition of pathogen adhesion by such nanoparticles is relevant to cancer therapy, it provided us with an idea to use the membrane of target bacteria to prepare specific nanoparticles that could compete with target bacteria to inhibit their adhesion and reduce their abundance, thereby improving anticancer efficacy. Preparing anticancer nanoformulations with certain components of microorganisms is also a promising research direction for cancer treatment. Yeast cell walls were used to create four different-sized nanoformulations that could remodel the immune microenvironment in tumors and tumor-draining lymph nodes, thereby suppressing tumor growth.^402^ Notably, due to the superiority of accumulating in tumor-draining lymph node, the T-cell-mediated anticancer immune response induced by the small size of the nano-formulation is stronger than the big size.^402^

#### Spore-based anticancer strategy

Spore is defined as a dormant or reproductive body produced by plants, fungi, and some microorganisms and it can develop into a new individual either directly or after fusion with another spore. In this article, spore refers specifically to the dormant body of bacteria and fungi.

One of the most common forms of spore-based strategy is drug delivery system. The dormant spores of *Bacillus cagulans*, a probiotic conducive to the treatment of intestinal inflammation and the regulation of gut microbial balance, can resist the harsh acidic environment, complex chemicals as well as temperature in gastrointestinal tract and germinate to probiotics under the activation by some nutrients in the gut.^403^ Additionally, during the process of germination, the hydrophobic protein coat on the surface of spores falls off.^404,405^ Thus, based on these physiological properties of the spores, a new oral drug delivery system for cancer therapy was developed by Song and colleagues.^403^ Specifically, the spore of *B. cagulans* was modified with DCA and loaded with chemotherapeutics, and the complex can disintegrate in the intestinal microenvironment, which is consequently accompanied by the self-assembly of nanoparticles containing chemotherapy drugs.^403^ More importantly, this system can protect the agents from acidic environment of the stomach, overcome intestinal barriers and decrease degradation of drugs in the epithelial cells, which would ultimately increase basolateral drug release into the circulation enhance and enhance the tumor inhibition efficency.^403^ Similarly, *Clostridium butyricum* spore was also used to develop oral drug delivery system for PDAC chemotherapy, which could markedly increase intratumoral drug accumulation.^406^

Additionally, spores of bacteria can also be used to treat cancer. Clostridial spores have been thoroughly investigated in that the obligate anaerobic nature of *Clostridium* makes them exclusively localized to and germinate in the necrotic/ hypoxic area of solid tumors.^407^ Thus, clostridial spores can be carriers of anticancer drugs or some special genes, which direct at TME. What’s more, clostridial spores can be used to decrease the side effects of chemotherapy. The toxicity of chemotherapy is mainly due to the lack of specificity for tumor cells and the damage to normal cells. Thus, genes expressing enzymes that convert the innocuous prodrug to toxic derivative can be introduced into clostridium, and injection of the transgenic bacterial spores can decrease systematic side effects when combined with nontoxic prodrug administration.^408^

## Cancer microbiota in clinical trials

Given the accumulating evidence involving the molecular mechanisms of microbiota effects on cancer development, an increasing number of clinical trials that aim to achieve clinical translation of microbial therapy are currently ongoing or have been completed,^70,371,373,409–412^ and some selected trials are summarized in Tables 2 and 3. Basically, there are two directions for the manipulation of the gut microbiota in cancer therapy: one is to boost therapeutic efficacy, and the other is to reduce therapy-related toxicity or side effects. For instance, a US trial (NCT04116775) for metastatic prostate cancer of FMT via endoscopy from pembrolizumab-sensitive participants into pembrolizumab-resistant participants aims to boost recipients’ antitumor efficacy and increase their tumor sensitivity to ICIs.^413^ Another clinical trial (NCT05032014) is assessing whether probiotics (Probio-49) can enhance PD-1 inhibitor efficacy in the treatment of liver cancer.^414^ To reduce immune-related toxicity, immunotherapy combined with FMT is being applied in renal cell carcinoma patients.^415^ Oral administration of probiotics is being used with concurrent pelvic chemoradiotherapy to evaluate its feasibility in the inhibition of radiation injury and related enteritis (NCT05032027).^416^ Another clinical trial actively is being performed to mitigate or prevent adverse events following chemotherapy with the use of probiotic supplements to keep breast cancer patients from experiencing chemotherapy-related toxicity.^373^ In addition, probiotic supplements can reduce and prevent the occurrence of chemotherapy-related cognitive impairment (CRCI).^373^ In addition, thyroid hormone withdrawal-related complications, such as dyslipidemia and constipation, in thyroid cancer patients following thyroidectomy could be alleviated by taking a probiotic complex.^417^ We are looking forward to seeing the prospective outcomes of these studies, which can be fundamental evidence for the clinical applications involving the gut microbiota, although further studies to determine the more specific and accurate molecular interactions underlying the microbiota and antitumor activity are still substantially needed.

**Table 2:** Selected clinical trials (ongoing) modulating the gut microbiota in cancer therapy

Trial ID	Offical Title	Research purposes	Cancer types	Microbial interventions	Primary Outcome Measures	Location
**Gut microbial modulation associated with anticancer therapeutic efficacy**						
**NCT04116775**^413^	A phase II Single Arm Study of Fecal Microbiota Transplant (FMT) in Men with Metastatic Castration Resistant Prostate Cancer Whose Cancer Has Not Responded to Enzalutamide + Pembrolizumab	To determine the anticancer effect of fecal microbiota transplant from participants who respond to pembrolizumab into those who have not responded in metastatic castration resistant prostate cancer	Prostate cancer	FMT via endoscopy	Percentage of participants with a PSA decline of ≥ 50% at any time point on study after FMT	America
**NCT05286294**^426^	MITRIC: Microbiota Transplant to Cancer Patients Who Have Failed Immunotherapy Using Faeces From Clincal Responders	To turn non-responders to immune checkpoint inhibitors into responders by modulating patients’ intestinal microbiota through FMT	Melanoma; head and neck squamous cell carcinoma; cutaneous squamous cell carcinoma; clear cell renal cell carcinoma	FMT	Objective Tumor Response Rate, incidence, nature, and severity of FMT-related Adverse Events	Norway
**NCT04951583**^427^	Phase II Trial of Fecal Microbial Transplantation in Patients With Advanced Non-Small Cell Lung Cancer and Melanoma Treated With Immune Checkpoint Inhibitors	To assess the impact of FMT on ICI response and survival	NSCLC, Melanoma and uveal melanoma	Investigational FMT capsules	Objective response rate in the NSCLC cohort	Canada
**NCT05032014**^414^	Probiotics Enhance the Treatment of PD-1 Inhibitors in Patients With Liver Cancer	To assess whether probiotics can improve the efficacy of ICI	Liver cancer	Probio-M9	Proportion of patients whose tumor volume shrinks to a predetermined value and maintains the minimum time limit	China
**NCT03829111**^428^	Pilot Study to Evaluate the Biologic Effect of CBM588 in Combination With Nivolumab/Ipilimumab for Patients With Metastatic Renal Cell Carcinoma	To determine the effect of *clostridium butyricum* CBM 588 probiotic strain (in combination with nivolumab/ipilimumab) on the gut microbiome in patients with metastatic renal cell carcinoma and evaluate the effect of CBM588 on the clinical efficacy of the nivolumab/ipilimumab combination	Renal cell carcinoma	CBM 588 probiotic strain	Change in *Bifidobacterium* composition of stool	America
**NCT05516641**^429^	Do Prebiotics Change Intestinal Biome in Rectal Cancer Patients Undergoing Neoadjuvant Therapy	To study microbiome modulating treatment could have an impact on CRC outcomes	Colorectal cancer	Dietary supplement: soluble corn fiber	Gut flora modulation	America
**NCT05083416**^430^	Effect of Prolonged Nightly Fasting (PNF) on Immunotherapy Treatment Outcomes in Patients With Advanced Head and Neck Cancer (HNSCC)-Role of Gut Microbiome	To evaluate if eating within an 8–10-h window during the day, without any caloric restriction, can lead to better response rates to immunotherapy in head and neck cancer patients	Head and neck cancer	Behavioral: prolonged nightly fasting	Rates of PNF compliance, change in gut microbiome and microbial metabolites	America
**Gut microbial modulation to prevent anticancer therapy-related toxicity/side effects**						
**NCT04038619**^431^	Fecal Microbiota Transplantation in Treating Immune-Checkpoint Inhibitor Induced-Diarrhea or Colitis in Genitourinary Cancer Patients	To assess the efficacy of FMT for clinical remission/response of immune-related diarrhea/colitis	Malignant genitourinary system neoplasm	FMT via colonoscopy	1. Incidence of FMT-related adverse events2. Clinical response/remission of immune-related diarrhea/colitis	America
**NCT04163289**^415^	Preventing Immune-Related Adverse Events in Renal Cell Carcinoma Patients Treated With Combination Immunotherapy Using Fecal Microbiota Transplantation	To study the safety of FMT combination immunotherapy treatment and reduce occurrence of immune-related toxicities in renal cell carcinoma patients	Renal cell carcinoma	FMT capsules	Occurence of grade 3 or higher immune-related colitis from the start of treatment with ipilimumab and nivolumab to 120 days after completion of treatment	Canada
**NCT05032027**^416^	A Randomized Controlled Clinical Study of Oral Probiotics on Radiation Enteritis Stage II Induced by Pelvic Concurrent Chemoradiotherapy	To determine if regulating intestinal tract flora will reduce the severity of radiation-induced mucositis in patients receiving pelvic radiotherapy	Pelvic cancer	Probiotic	The incidence of grade 3 enteritis	China
**NCT04869956**^432^	Gut Microbiome Modification Through Dietary Intervention in Patients With Colorectal Cancer: Response to Surgery	To assess the effect of a dietary fiber diet on the prognosis of colorectal cancer patients following surgery	Colorectal cancer	Dietary intervention: a high-fiber diet rich in polyunsaturated fatty acid	Rate of anastomotic leakage surgical site infection	Spain

**Table 3:** Selected clinical trials (completed) modulating the gut microbiota in cancer therapy

Trial ID	Offical Title	Cancer types	Microbial interventions	Primary Outcome Measures	Location
**Gut microbial modulation associated with anticancer therapeutic efficacy**					
**NCT03358511**^433^	Engineering Gut Microbiome to Target Breast Cancer	Breast cancer	Probiotic	Mean number of cytotoxic T lymphocytes (CD8 + cells)	America
**NCT03290651**^434^	Re-setting the Breast Microbiome to Lower Inflammation and Risk of Cancer	Breast cancer	Probiotic natural health product: RepHresh Pro-B	Change in breast microbiota	Canada
**NCT03072641**^435^	Using Probiotics to Reactivate Tumor Suppressor Genes in Colon Cancer	Colon cancer	ProBion clinica	Changes in microbiota composition after probiotics use	Sweden
**Gut microbial modulation to prevent anticancer therapy-related toxicity/side effects**					
**ChiCTR-INQ-17014181**^436^	The effect of Probiotics on preventing patients with breast cancer from cancer-related cognitive impairmentand and its mechanism	Breast cancer	Probiotic capsules with *Bifidobacterium longum*, *Lactobacillus acidophilus* and *Enterococcus faecalis*	Incidence of cancer-related cognitive impairment	China
**NCT01410955**^437^	Prevention of Irinotecan Induced Diarrhea by Probiotics. A Phase III Study	Colorectal cancer	Probiotic	Prevention of grade 3–4 diarrhea by probiotics in patients treated with irinotecan-based chemotherapy	Slovakia
**NCT01839721**^438^	Impact of Probiotic BIFILACT® on Diarrhea in Patients Treated With Pelvic Radiation	Prostate cancer; gynecologic cancers	Probiotic: Bifilact®	The efficacy of probiotic Bifilact®	Canada
**NCT04040712**^439^	Fecal Microbiota Transplantation to Treat Diarrhea Induced by Tyrosine-kinase Inhibitors in Patients With Metastatic Renal Cell Carcinoma: a Randomized Clinical Trial	Renal cell cancer	FMT	Rate of patients who experience resolution of diarrhea 4 weeks after the end of treatments	Italy
**NCT03420443**^440^	Randomized Clinical Trial of Effects of Synbiotics on Intestinal Microbiota in Patients Undergoing Short-course Preoperative Radiotherapy During Treatment of Rectal Cancer	Rectal cancer	Dietary Supplement: Oat bran and blueberry husks	Action of synbiotics on irradiated GI mucosa in rectal cancer treatment	Sweden
**NCT01549782**^441^	Effect of a Mixture of Inulin and Fructo-oligosaccharide on Lactobacillus and Bifidobacterium Intestinal Microbiota of Patients Receiving Radiotherapy: a Randomised, Double-blind, Placebo-controlled Trial	Endometrial neoplasms	Dietary Supplement: Inulin, Fructo-oligosaccharide and Maltodextrine	Changes in Lactobacillus and Bifidobacterium populations	Spain

## Future challenges and outlook

Therapeutic resistance and adverse effect are still the main obstacles in the management of cancer treatment, despite great efforts to optimize therapeutic effects and minimize adverse toxicity.^418^ Hence, the future of utilizing the cancer-associated microbes in the clinic is not devoid of prospects and challenges that should be recognized and addressed.

Currently, due to lacks of uniformed methodology, including differences in sample selection and collection, technology, data quality as well as resource analysis, the homogeneity and consistency of mechanistic understanding of microbial effect on cancer could not be ensured. Different samples from same subjects may lead to largely heterogeneous results. For instance, the composition and richness of microbiota colonized on the digestive tract mucosa and those in feces are similar, but not identical.^419^ If only one type of sample is included in the study, it may lead to biased results. Henceforth, several different types of samples should be collected and investigated for objective research results.

In addition, errors may occur in the process of sample collection and handling. Because of the low biomass of tumor microbiota, any contamination of samples would dramatically hamper the microbial research, which can be caused by long surgery, cross-contamination from other samples and complex environment in laboratory.^32,420^ Thus, in order to ensure the rigorous research results, it is crucial to implement multiple measures to decrease the possibility of sample contamination, such as wearing clean protective clothing to cover all exposed human surfaces when collecting sample.^420^ In addition, technical variables, e.g., sample handling, DNA extraction, bioinformatics, and data acquisition, exist in identifying specific compositional and functional microbial signatures. Some may use 16 S rRNA sequencing of saliva or bile samples, while others may examine stool samples, and high-throughput data may be produced either by next-generation sequencing (NGS) or third-generation sequencing (Nanopore or SMRT sequencing), increasing the heterogeneity of data resources and accessibility difficulties. To address these challenges, a comprehensive “standard operation procedure (SOP)” for all methodologies could be introduced in coming years, with the precondition of reaching consensuses on sample collection, technique selection, and data sharing and analysis, despite the multiple existing protocols proposed by the Microbiome Quality Control (MBQC) project consortium.^421^

In addition to the methodological challenges described above, individual biological differences may encumber the application of microbial strategies. Various factors, including genetics, diet habits, age, sex, accompanying diseases and regional variations,^422,423^ can influence the features of human microbiota. He and colleagues have shown that host location has the strongest impact on gut microbiota variations compared to other factors, which is marked by the large variations in the abundance of Firmicutes among populations in different districts of Guangdong, China.^424^ This regional variation to human microbiota is one of main reasons limiting the spread and application of certain findings in other districts.^424^ For example, some microbes can serve as non-invasive diagnostic biomarkers of CRC,^425^ but it may suffer a setback when this finding is generalized to other regions.^424^ It must be noted that, from a deeper perspective, region is not a single factor, but a complex of various factors, including economic development, diet habit, ecological environment in the region and etc. Thus, it is not surprising that geography has such a noticeable influence on the human microbiota.

In the near future, emphasis should be placed on distinct microbiota stratification, and the unique strain obtained in different hosts might be researched by a “single microbe” profile, analogous to the single-cell sequence, which can facilitate the accurate capture of precise impacting mechanisms. Furthermore, some special preclinical models, e.g., patient-derived organotypic tumor spheroids in short-term 3D cultures within the same environmental surroundings, can be used to validate the findings in vitro and to link the molecular mechanisms to applications.

Despite accumulating evidence observed in human subjects, the corresponding clinical interventions targeted at microbes have yet to be translated to mature applications for cancer patients. The causes resulting in this phenomenon are extremely complex, which can be partly attributed to the individual differences in sensitivity to the same microbial agents. Can microbes-targeted interventions be integrated in existing cancer management system to exert more comprehensive and favorable antitumor effects? The problem remains unresolved till now and thus more preclinical research and prospective clinical trials are needed to figure out the challenges.

Finally, although many challenges remain for now, the great importance and full potential of gut microbiota cannot be overstated for the development of new anti-cancer strategies, and it is necessary to explore a holistic approach that incorporates microbial modulation therapy in the current cancer management system.
